# Effects of Dominance and Diversity on Productivity along Ellenberg's Experimental Water Table Gradients

**DOI:** 10.1371/journal.pone.0043358

**Published:** 2012-09-12

**Authors:** Andy Hector, Stefanie von Felten, Yann Hautier, Maja Weilenmann, Helge Bruelheide

**Affiliations:** 1 Institute of Evolutionary Biology and Environmental Studies, University of Zurich, Zurich, Switzerland; 2 Microsoft Research, Cambridge, United Kingdom; 3 Institute of Biology/Geobotany and Botanical Garden, Martin Luther University Halle-Wittenberg, Halle (Saale), Germany; University of San Diego, United States of America

## Abstract

Heinz Ellenberg's historically important work on changes in the abundances of a community of grass species growing along experimental gradients of water table depth has played an important role in helping to identify the hydrological niches of plant species in wet meadows. We present a previously unpublished complete version of Ellenberg's dataset from the 1950s together with the results of a series of modern statistical analyses testing for hypothesized overyielding of aboveground net primary production as a consequence of resource-based niche differentiation. Interactions of species with water table depth and soil type in the results of our analyses are qualitatively consistent with earlier interpretations of evidence for differences in the fundamental and realized niches of species. *Arrhenatherum elatius* tended to dominate communities and this effect was generally positively related to increasing water table depth. There was little overyielding of aboveground net primary production during the two repeats of the experiment conducted in successive single growing seasons. Examination of how the effects of biodiversity on ecosystem processes vary across environmental gradients is an underutilized approach – particularly where the gradient is thought to be an axis of niche differentiation as is the case with water availability. Furthermore, advances in ecology and statistics during the 60 years since Ellenberg's classic experiment was performed suggest that it may be worth repeating over a longer duration and with modern experimental design and methodologies.

## Introduction

There is a long tradition in ecology of investigating interactions by growing species alone and in competition with others. In this article we present a previously unpublished complete dataset from a classic example of this type of experiment: Heinz Ellenberg's “Hohenheim groundwater table experiment” on the effects of water table depth on communities of grassland plant species grown in monoculture and mixture [1], [2]. In plant ecology this monoculture *vs.* mixture approach has been used to investigate competition between species and the consequences of species interactions for ecosystem primary productivity [3]–[8]. Our paper therefore also presents the results of a contemporary analysis of overyielding and the effects of diversity on productivity using Ellenberg's data and additive partitioning methods [6], [9].

**Figure 1:**
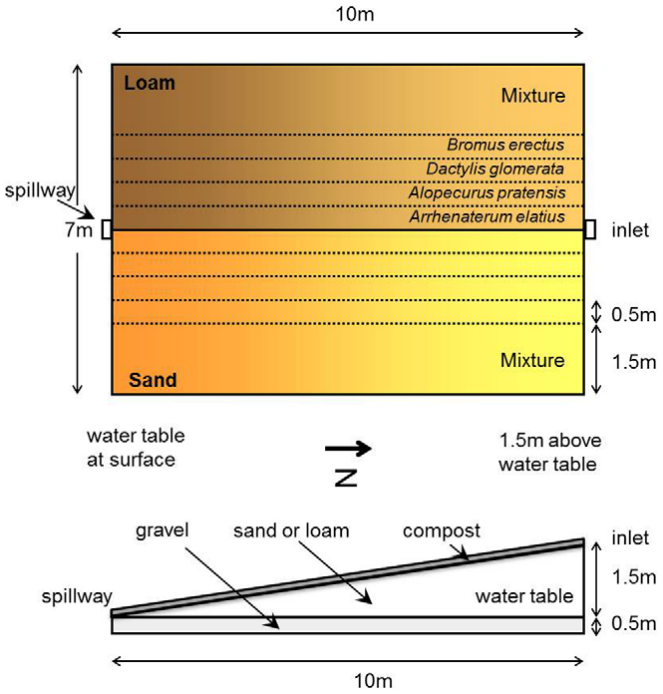
Schematic diagram of Ellenberg's water table depth gradient experiment. The diagram shows the concrete tank from above (top) and the side (below) following Ellenberg (1953, 1954) and Schulze and Beck (2002). Note that the compost layer is on top of the sand, not underneath it as shown in Schulze and Beck (2002). The concrete tank was divided into two halves filled to varying depth with either loam or sand to generate increasing distance to the water table. Each half was divided into strips sown either as monocultures of individual species (4 in 1953, as shown here, 6 in 1952, see Figure S1) or a mixtures of all species combined.

Heinz Ellenberg is well known for having introduced the concept of indicator values, based on the occurrence of species along gradients in nutrient and water supply, pH and climate and other environmental variables [10]. What is less well known is that his concept of fundamental and realized niches predated the frequently cited paper of Hutchinson [11] and was derived from grass communities grown along experimental gradients of depth to the water table [1], [2]. Ellenberg created the experimental gradients using a concrete tank with sides that gradually increased in height from one end to the other (Fig. 1). The tank was filled with soil that also varied in depth along its length by following the height of the walls. Water flowed through the tank from an inlet at the deep end to a spill way and outlet at the shallow end to produce a gradient of depth to the water table. As a supplement to our paper we present a newly discovered complete version of Ellenberg's data from his Hohenheim experiment [1], [2].

Although Ellenberg's Hohenheim experiments date from the early 1950s they more recently played an important role in defining the hydrological niches of plants in wet meadows in S.W. England. Silvertown and colleagues [12] did this using data on species occurrence in the Somerset Levels in relation to the depth of the water table (estimated using bore hole measurements). Randomisation tests of the relative abundance of species from Ellenberg's published Hohenheim data showed that the fundamental niches of species, as measured in monoculture, overlapped far more than expected by chance and more than the realized niches of plants grown together in mixed communities. The first result suggests that, when grown alone, species tend to favour the same conditions (have similar fundamental niches), while the second result suggests that competition drives species to have different realized niches [12].

The aim of Ellenberg's experiments was to create a gradient in soil moisture - a resource-based potential niche axis. Coexistence of species through resource partitioning is generally expected to result in overyielding of community productivity. We recognized that Ellenberg's species abundance data in monoculture and mixture could also be used to test for overyielding of aboveground biomass production along the gradient in depth to the water table. At first it appeared that this would not be possible since Ellenberg's [1], [2] published data does not include matched values for the aboveground biomass of species in both monoculture and mixtures as required by most tests of overyielding. However, on investigation, we were able to retrieve the raw data from Ellenberg's hand-written notes that were needed to complete the dataset and to calculate the measures of complementarity necessary to test for the overyielding hypothesized to result if species were differentiated with respect to a resource-based niche, in this case the gradient of depth to the water table.

## Materials and Methods

### Data

The complete dataset comprises measurements taken from two similar experiments conducted in 1952 [1] and 1953 [2]. The 1952 experiment involved six species (*Poa palustris*, *Festuca pratensis*, *Alopecurus*, *pratensis*, *Dactylis glomerata*, *Arrhenatherum elatius*, *Bromus erectus*; Fig. S1) while the 1953 work involved a subset of four (Fig. 1). In both years species were sown to achieve approximately equal seedling density.

The experiments were carried out in one large concrete tank of 7 m×10 m, divided into two halves of 3.5 m×10 m with one side filled with silty loam, the other side with sand ([13], Fig. 1). At the shallow end the tank was 50 cm deep and at its deep end it was 2 m deep. The gradient in water table depth was created by filling the tanks to create an inclined soil surface, ascending from 50 cm to 200 cm above the bottom of the concrete container. Water was passed through the tank so that it was approximately at ground level at the shallow end of the tank (in 1953 the target was to have the water table exactly at the soil surface while in 1952 the soil was slightly below the level of the tank causing 5 cm of standing water above the soil surface) and 140 cm below the soil surface at the deep end of the tank (the water table was apparently raised by 5–10 cm at the deep end of the tank relative to the shallow presumably due to capillary forces). The two years also differed in the accuracy with which the water table was maintained: in 1952 fluctuations of several decimeters occurred, whereas by 1953 the water table was kept constant through a more continuous inflow and outflow of water.

The experiments were carried out on strips running along the water gradient. The width of the strips differed between 1952 and 1953 because of the different number of species grown in the two years and the fixed 7 m width of the concrete tank. Knowledge of the size of these experimental units is essential for translating the relative yield values reported by Ellenberg [1], [2] into biomass per unit area, which had not been published by Ellenberg (Supporting Information S1). The strip width given for 1953 [2] is 0.5 m for the four species when grown alone and 1.5 m for the mixtures of all four species combined (correctly giving a width for each half of the tank of 3.5 m). In 1952, a further two species were grown, bringing the total to six (Fig. S1). The width of the mixture strip was reduced to1.2 m leaving 2.3 m in each half for the monocultures [1]. Assuming each of the six monocultures is grown on a strip of equal width produces a value of 0.38 m. These dimensions were checked against historic photographs of the experiment (Supporting Information S2) and were confirmed with certainty because they correctly reproduce all individual data values given in Ellenberg's papers and notes.

The strips were separated belowground by panels to exclude root interference from adjacent strips (the thickness of these internal walls is not given in Ellenberg [1], [2] and Lieth and Ellenberg [13] and since they would reduce the width of each strip by a negligible amount in any case our calculations and the measurements we report (e.g. Fig. 1) do not take them into account). The gradient in water depth was divided in 10 (in 1952) or 11 (in 1953) sections, in which biomass was harvested, either from a species growing alone (monocultures) or in the mixed communities produced by combining the four species grown in 1952 or the six species the following year. Three samples along the gradient were oven-dried, and using these samples, fresh weight was related to dry weight. In 1952, some species in monocultures did not reach complete coverage in some sections due to poor germination (even though the median cover values were high see Fig. S2 and Table S1). Ellenberg predicted what the fresh weight of each species would have been assuming complete 100% cover (e.g. if a species achieved only 50% cover its biomass value would be doubled). Such cover values were not provided in the manuscript for 1953 suggesting that full cover was approximately attained for all species, which is corroborated by photographs of that year (Supporting Information S2). Thus, the values for 1952 were cover-adjusted, while those from 1953 were almost certainly not. For comparability with the earlier works of Ellenberg and colleagues and Silvertown et al., we analyse the data in the same form as in these earlier works, but an open access version of the full dataset is provided as supplementary material to allow further analyses of this historically important data (Supporting Information S3, S4 and S5).

In total the newly assembled complete dataset comprises 416 values (6 species×10 levels of water table depth in 1952, +4 species×11 levels of water table depth in 1953, ×2 soil types, ×2 levels of plant diversity).

### Measures of overyielding

As explained in the introduction, Ellenberg's data is thought to demonstrate evidence for hydrological niches and resource-based niches of this type are thought to result in overyielding. Our analyses therefore tests whether these hydrological niches along a resource gradient result in overyielding of productivity and how this relationship was affected by differences in soil type and year. For this reason, and since the number of species varied between 4 and 6 in the two different years of the experiment, we concentrated on analyses of community- and ecosystem-level responses, namely additive partitioning of biodiversity effects [6], [9] and relative yields and relative yield totals [5]. The additive partitioning of biodiversity effects produces absolute measures while relative yields are, as the name suggests, a relative measure. For brevity, the main text presents complementarity and selection effects [6]. Dominance, trait-dependent and trait-independent complementarity effects from the tripartite additive partitioning [9], [14], [15] are presented as supplementary material but don't appear to bring any further insights (Fig. S3 and S4). The selection effect examines the covariation between changes in the deviations from expected relative yields of species in mixture and their productivity in monoculture: positive selection effects occur when, on average, species with higher than average monoculture biomass increase their relative abundance in communities while negative selection effects occur when species with lower than average monoculture biomass increase their relative abundance in mixture. Complementarity effects occur when the total biomass of a mixture is more (or less) than expected based on the monoculture yields, producing overyielding (or underyielding). Positive complementarity effects (or overyielding) occur when increases in the biomass of some species are not exactly compensated by decreases in the biomasses of others. This could occur due to complementary resource use, a reduction of natural enemy effects in mixtures, some form of facilitation or some combination of these effects. Negative complementarity effects are consistent with some type of interference competition (although the biological details remain unknown). The relative yield of a species is its biomass in mixture expressed as a proportion of its yield in monoculture. The relative yield total (RYT) of a mixture is the sum of the individual relative yields. Values of RYT greater than one indicate overyielding (equivalent to positive complementarity effect values). Further explanation and comparison of the overyielding methods can be found in [15]. Details of the overyielding calculations are given in the supplementary material (Supporting Information S6).

### Analysis

We modelled changes in these response variables across the gradients in depth to the water table using regressions that included linear and quadratic terms only (depth and depth squared) because of the limited size of the dataset and because while it seemed plausible that maximum or minimum values of the responses could occur at intermediate water table depths, we had no *a priori* biological basis for allowing more complex relationships in monoculture.

Competitive displacement of species from the middle of the gradient to both higher and lower water table depths could produce bimodal relationships in mixtures [16] but exploratory generalized additive models for the aboveground biomass yields of the species in mixture provided no support for a higher degree of complexity than the quadratic relationship (Fig. S5). We therefore limited ourselves to the simpler quadratic models for consistency across monocultures and mixtures and due to risk of over-fitting complex higher-order polynomials to this limited dataset without supporting evidence.

We then tested whether the resulting curves varied with soil type and year using linear mixed-effects models containing linear and quadratic terms for water table depth and including a random factor (“gradient”) with four levels, one for each soil type in each year, to reflect the grouping of the data into two years and the splitting of the concrete tank into two halves. For the biodiversity effects (trait-dependent and trait-independent complementarity, selection and dominance effects), the variance was not constant, so we transformed the values by taking the square root of the absolute values and restoring the original positive or negative sign [6]. The analyses were performed in R 2.12.1 [17] using the lmer function from the lme4 library [18] for both the species- and community-level data (necessary due to crossed random effects for the species-level data where species repeat in the different year and soil type combinations). For the species-level data, we considered fixed effects of water table depth, soil type and species while treating the halves of the concrete tank, the strips and the species compositions (6 monocultures plus 2 mixtures) as random effects. For the community level data, we considered fixed effects of water table depth, soil type and year while treating the four gradients (produced by the two halves of the concrete tank in each of the two years) as random effects. Following examples given by the software authors [19], our analysis took a model simplification approach to identifying the single most parsimonious model from the series of nested models (i.e. the one with the lowest BIC, favouring simpler models when pairs of nested models were tied with BICs within 2 units per difference in the number of parameters). We used the BIC since it is one of the more widely used information criteria and there is less risk of over-fitting unnecessarily complex models to small datasets than with the AIC. In the supplementary material we also provide graphs of the full quadratic models for each variable.

## Results

**Figure 2:**
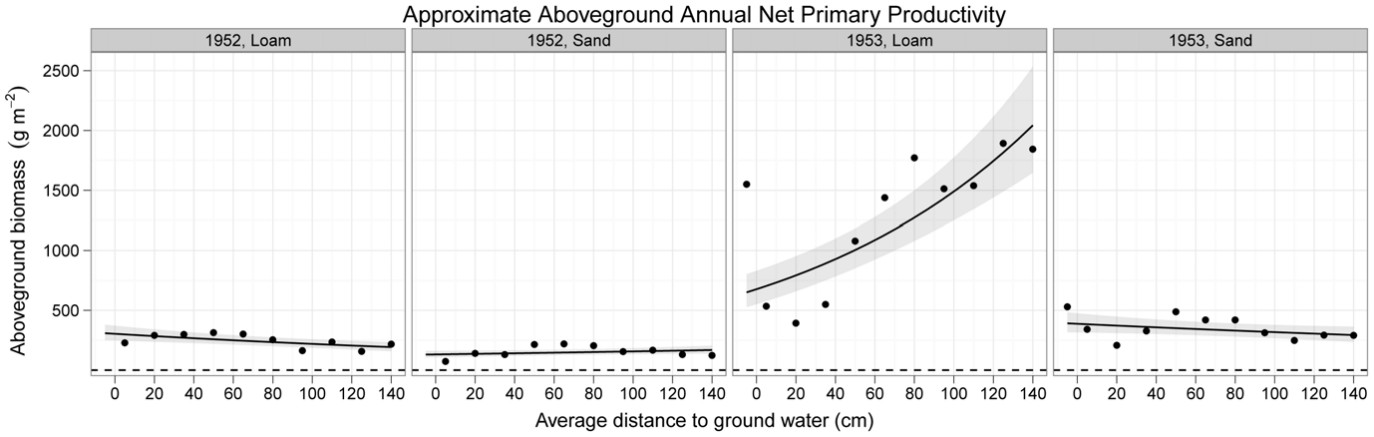
Results of the mixed-effects model analysis of the effect of the water table depth on total aboveground biomass production on sand and loam soils in 1952 and 1953. Note that there is little evidence for limitation of production by water: no drought was imposed. On the contrary, in 1953 there may have been some water logging on the loam since conditions were more productive as depth to the water table increased. The lines are slopes from the mixed-effects model with their 95% confidence intervals (shaded).

The aim of Ellenberg's experiments was to create wetter and drier growing conditions by varying depth of the water table on two soils of different water-holding capacity in order to look for evidence for what he coined the terms physiological and ecological behaviour and what we would now call fundamental and realized hydrological niches, respectively (Fig. 1). Interestingly, little previous assessment has been made of the success of this manipulation. Counter to our expectations there is very little evidence for any effect of drought on total aboveground net biomass production (Fig. 2). In fact, the most pronounced effect was of increasing biomass with greater depth to the water table on the loam in 1953, suggesting not drought but rather water-logging on this soil type in this wet year (Fig. 2; Table S2).

**Figure 3:**
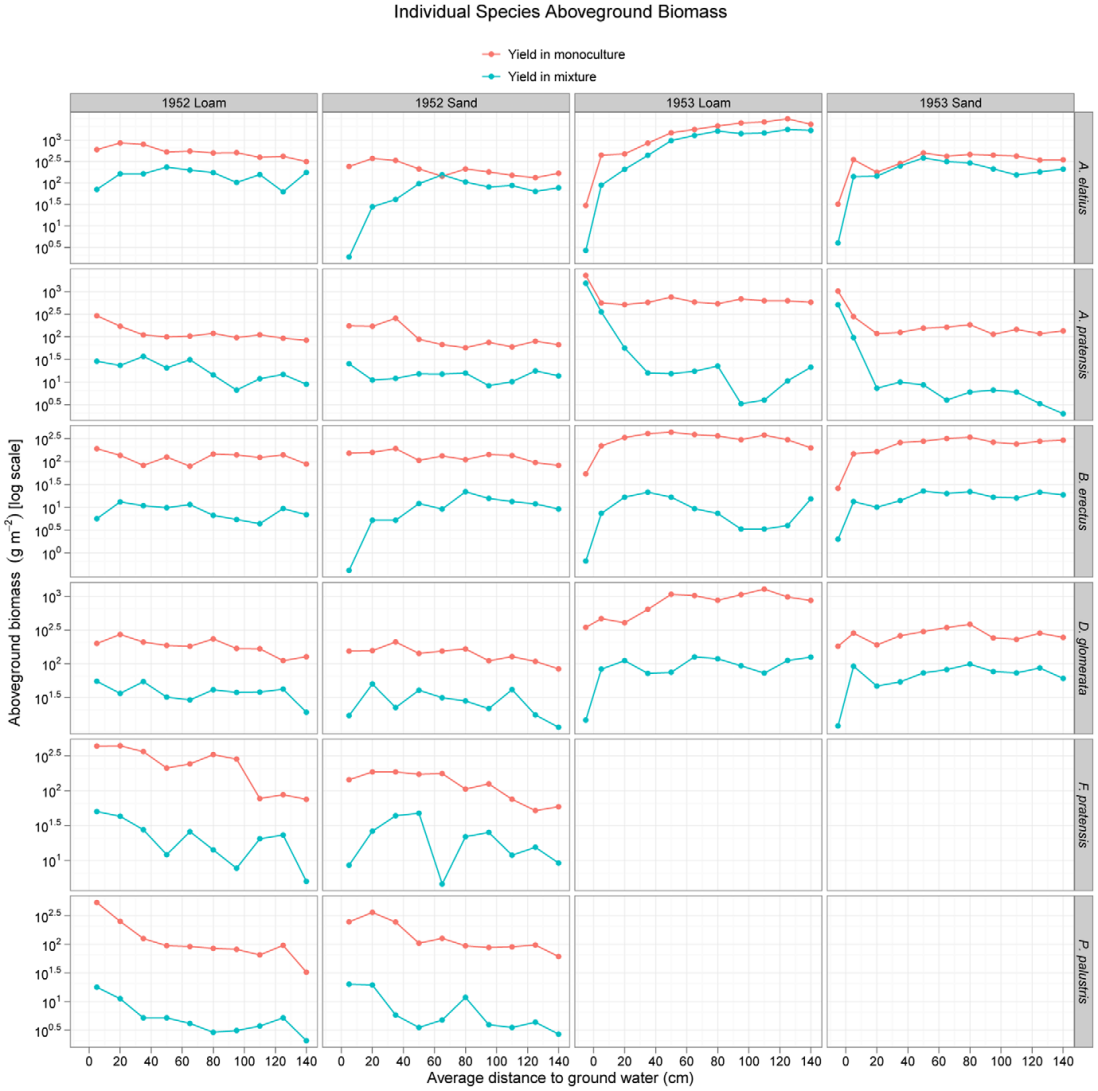
The complete dataset showing the yields of six different species in monoculture and mixture across the water table depth gradient on two soil types in two years. Note the absence of two species in 1953.

**Figure 4:**
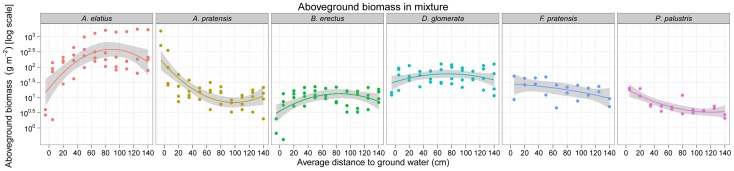
Results of the mixed-effects model analysis of the yields of individual species across the experimental water table depth gradient. Undetectable interactions with soil type have been removed from the model while the results are essentially averaged across the two years by including year as a random factor. The curves are slopes from the mixed-effects model with their 95% confidence intervals (shaded). Note that *F. pratensis* and *P. palustris* were present only in 1952.

The newly assembled complete dataset of the mass of the different species grown in monoculture and mixture across the water table depth gradients on the two soil types in the two years is shown in Figure 3 and Figure S6. Ellenberg [1], [2] interpreted subsets of these data as demonstrating differences in the fundamental and realized niches of species, a conclusion supported by the randomisation tests of species relative (percent) abundances reported by Silvertown et al. [12]. The results of our analysis of the full data on the yields of the species in monoculture are also consistent with differences in fundamental niches via the presence of many interactions between species identity and the environmental variables (Table S3), while the same analysis of relative yields (Fig. S7; Table S4) of individual species in mixture is similarly consistent with niche differences. Model simplification for the aboveground biomass yields of the species in mixture selected a model with different quadratic regressions for each species but which omitted interactions with soil type (Fig. 4; Table S3). *Arrhenatherum elatius* showed a positive convex relationship with increasing depth to the water table as did, to a lesser extent, *Bromus erectus and Festuca pratensis*. In contrast, *Alopecurus pratensis* showed a negative concave relationship, as did *Poa palustris* (but with less curvature). *Dactylis glomerata* yields peaked at the middle of the gradient but were fairly insensitive to the water table depth.

Relative yields of individual species showed a similar but slightly more complex response with species having different curvatures (quadratic relationships) of their responses to depth to the water table and with soil type (but no 3-way interaction between soil, species and water table; Fig. S8; Table S4). Comparing observed with expected relative yields (where expected relative yields are simply 1/S where S is the number of species in the mixture: 1/6 in 1952 and 1/4 in 1953) showed that *Arrhenatherum elatius* was the species with the highest gains relative to expected values across most of the water table gradient in both years and on both soil types (Fig. S7). The performance of *Arrhenatherum* and *Alopecurus* appears to be somewhat negatively related since where *Arrhenatherum* performed better than expected across most of the gradient, *Alopecurus* under-performed. However, *Arrhenatherum* did only as well as, or worse than expected when the water table was high, conditions where *Alopecurus pratensis* did as well or better than expected. In contrast, *Dactylis glomerata* was fairly unresponsive: it did about as well as expected under all conditions. *Bromus erectus* generally performed worse than expected across the entire gradient.

**Figure 5:**
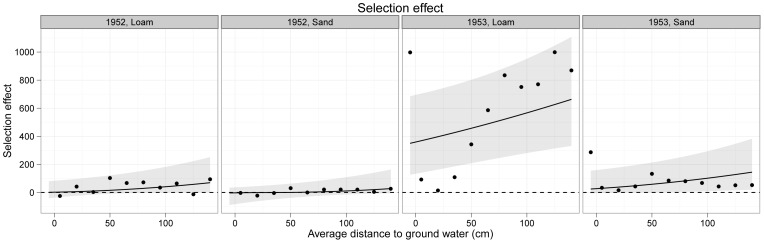
Results of the analysis of the effect of the water table depth on the strength and direction of the Selection Effect on sand and loam soils in 1952 and 1953. The curves are back-transformed slopes from the mixed-effects model with their 95% confidence intervals (shaded). The positive Selection Effect on loam in 1953 is driven by increasing dominance by *Arrhenatherum elatius* as depth to the water table increased.

Selection effects were strongest on loam in 1953 where they were increasingly positive as depth to the water table increased (Fig. 5). This was largely due to the dominance of *Arrhenatherum elatius*, a species with a relatively high monoculture biomass (Fig. 3), as described above.

**Figure 6:**
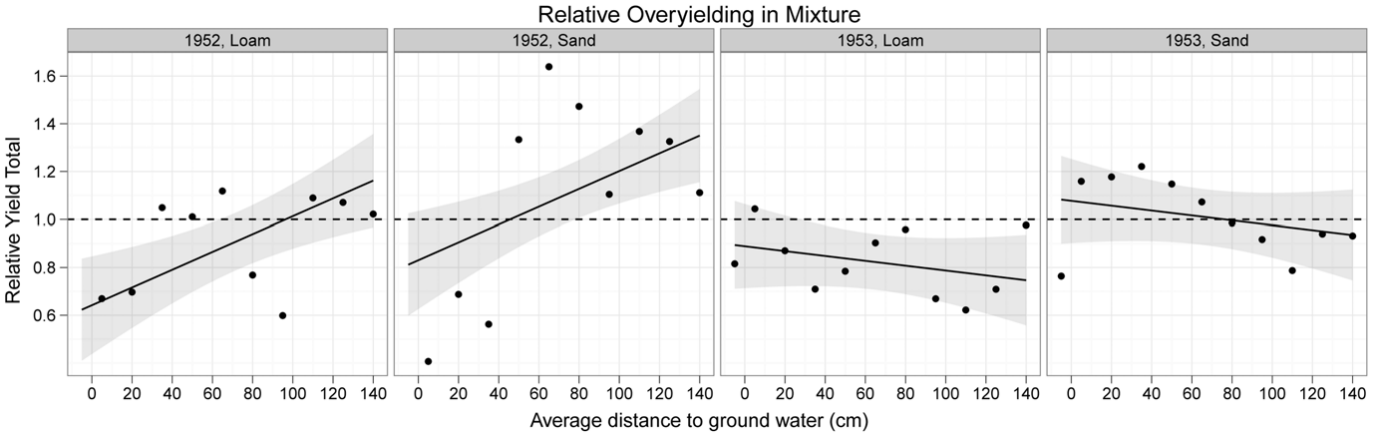
Results of the analysis of the effect of the water table depth on relative overyielding (RYT) on sand and loam soils in 1952 and 1953. The curves are back-transformed slopes from the mixed-effects model with their 95% confidence intervals (shaded). The dotted lines show the zero sum null expectation where species perform the same in mixtures as in monoculture and increases in some species populations are exactly offset by declines in others. Complementarity effects produced similar results but the data are not so well behaved (Figure S9).

There was little evidence for complementarity (Fig. S9): confidence intervals for average relative yield totals contained zero in most cases with some overyielding at greater water table depths in 1952 and underyielding on loam in 1953 when *Arrhenatherum* strongly dominated and drove a positive selection effect (Fig. S8). Since RYTs and complementarity effects are linearly related [6] they show similar patterns (Fig. 6) but the complementarity effect data were statistically less well behaved.

## Discussion

Ellenberg's experimental manipulation of water table depth has been highly influential work, both in its original publication and through later use, notably by Silvertown and colleagues to support their demonstration of hydrological niches in wet meadow communities [12]. The results of our analysis seem consistent with this earlier work: the many species-by-environment interactions in the analysis of both the monoculture and mixture data are consistent with differences in the fundamental and realized niches of these grass species. However, we found limited evidence of overyielding.

The limited evidence for positive complementarity effects in Ellenberg's data is in some ways surprising given that the same information has been used as evidence for hydrological niche differences and that resource-based niches are thought to lead to overyielding. We can think of three possible explanations for their absence. First, although previous analyses by Ellenberg and Silvertown and colleagues are consistent with species coexistence they do not conclusively demonstrate that stabilizing differences between species are strong enough to overcome any differences in fitness to ensure stable long-term coexistence. Rather, it could be that in the long-term there would have been competitive exclusion with different species dominating at the points on the water table gradient where they were competitively superior. Second, theory predicts that there are mechanism for the stable coexistence of species without positive effects on community productivity [20] and it is possible that hydrological niches are one such example (although this seems unlikely given that that the published example of a coexistence mechanism that does not lead to overyielding is the competition-colonisation trade off which is not a resource-based niche like the hydrological gradient examined here). Third, the lack of complementarity effects may simply reflect the short duration of the two single year experiments and insufficient time for overyielding to fully emerge. A meta-analysis of 47 biodiversity experiments [3] found that complementarity effects were initially weak and increased over time and were still linearly increasing even after several years. Testing these alternative hypotheses would require repeating Ellenberg's experiments (with modern improvements in design and methodology) and running them for a longer duration, ideally beyond the range of recent studies [3] (5–10 years or more). Finally, while overyielding is consistent with many forms of stable coexistence through niche differences (but see [20]) it may be an imperfect measure that may also reflect other processes [21], [22].

We were also surprised by another aspect of the data. We had the impression that the water table depth gradients were likely to impose drought, at least on some species and particularly on the freely draining sand. However, there is little evidence for a drought effect. On the contrary, on the loam in 1953 aboveground biomass production increased with depth to the water table to very high levels, suggesting if anything the opposite: limitation of production by water logging as the water table approached the soil surface that could be due to the particularly wet summer of 1953 exacerbated by the low oxygen supply on loam compared to sand (Fig. S10). Whether such high productivities would be repeatable is not clear and is another incentive to repeat Ellenberg's experiments with modern improvements. In addition to improved replication and randomisation, these future studies should incorporate measurements of actual water table depths and soil water potential and could then attempt to impose drought at the drier end of the gradient.

Ellenberg's data show evidence for strong positive selection effects on the more fertile loam soil during 1953. Positive selection effects occur when species with higher-than-average monoculture aboveground biomasses dominate mixtures: in this case *Arrhenatherum* mainly drove the effect. Furthermore, this effect was generally positively related to increasing depth to the water table. While we cannot know the biological mechanisms underlying the positive selection effects, one simple explanation is that species with high aboveground biomass in monoculture also had high belowground biomass enabling them to better access the deeper water table. Both *Festuca* and *Poa* showed declining biomasses with increasing depth to the water table, as did *Alopecurus* in 1953, although only when in competition with other species in the mixture (particularly *Arrhenatherum*).

Not surprisingly, developments in experimental design and statistical analysis since the 1950s mean that Ellenberg's experiments could be improved in several ways if repeated. Lack of true replication in Ellenberg's experiments means that we cannot know how repeatable the yields at a given point on the gradient of water table depth on a particular soil and in a particular year are, and an increase in replication would therefore be an essential feature of a repeat study. The experiments also examine only one set of species grown alone and when combined together. Replicate species pools and mixtures of intermediate diversity - particularly species pairs - would also be useful for generalizing the results and for investigating species interactions in greater detail. The spatial arrangement of the strips also has the (wider) mixed community strips always at the edge of the concrete tanks (Fig. 1). Replicate monocultures and mixtures could be randomised to avoid potential edge effects or, if replication were low, they could be systematically arranged so that equal proportions of the monoculture and mixture strips were at the edge. We hope this article will stimulate these updated repeats of Ellenberg's experiments in order to further the experimental investigation of the hydrological niche and its effects of productivity and ecosystem functioning.

## Supporting Information

Supporting Information S1 **Ellenberg's adjustment for incomplete cover.**
(DOCX)

Supporting Information S2 **Historical photographs of the Hohenheim-Experiment.**
(PDF)

Supporting Information S3 **Metadata.**
(DOCX)

Supporting Information S4 **Ellenberg's full data set.**
(TXT)

Supporting Information S5 **Ellenberg's climate data set from the weather station in Hohenheim.**
(TXT)

Supporting Information S6 **Additive partitioning of biodiversity effects.**
(DOCX)

Figure S1 **Schematic diagram of Ellenberg's water table depth gradient experiment in 1952.**
(TIF)

Figure S2 **Means biomass for the monocultures of the 6 species used in the 1952 run of the experiment.** Green symbols show Ellenberg's data as adjusted for cover levels below 100% and red symbols show the same mean values allowing for the observed level of cover (i.e. undoing Ellenberg's changes).
(TIF)

Figure S3 **Results** **of the analysis of the effect of the water table depth on the strength and direction of the trait-dependent complementarity effect on sand and loam soils in 1952 and 1953.** The lines are slopes from the mixed-effects model with their 95% confidence intervals (shaded).
(TIF)

Figure S4 **Results** **of the analysis of the effect of the water table depth on the strength and direction of dominance effect on sand and loam soils in 1952 and 1953.** The lines are slopes from the mixed-effects model with their 95% confidence intervals (shaded).
(TIF)

Figure S5 **Estimated smoothing curves for the yields of individual species across the experimental water table depth gradient.** The solid line is the smoother and the dotted lines are 95% point-wise confidence bands. Note that the smoother is centred around zero.
(TIF)

Figure S6 **Dry biomass.** Aboveground biomass of species in monoculture (blue) and mixture (green) on the two soil types in the two experimental years together with the total community aboveground biomass (black). Note that the black symbols within each column of panels are the same and are the sum of the green symbols.
(TIF)

Figure S7 **Relative yields of six different species across the water table depth gradient on two soil types in two years.** Note the absence of two species in 1953.
(TIF)

Figure S8 **Results** **of the mixed-effects model analysis of the relative yields of individual species across the experimental water table depth gradient.** The curves are back-transformed slopes from the mixed-effects model with their 95% confidence intervals (shaded).
(TIF)

Figure S9 **Results** **of the analysis of the effect of the water table depth on the strength and direction of the trait-independent complementarity effect on sand and loam soils in 1952 and 1953.** The curves are back-transformed slopes from the mixed-effects model with their 95% confidence intervals (shaded). The negative Complementarity Effect on loam in 1953 is mainly driven by increasing dominance by Arrhenatherum elatius as depth to the water table increased.
(TIF)

Figure S10 **The climate data from the weather station in Hohenheim clearly showing that 1953 had a very wet summer.**
(TIF)

Table S1 **Available cover data of the monocultures in 1952 with median cover, cover corrected biomass mean (se) in g and the uncorrected biomass mean (se) in g within species and by soil type.**
(DOC)

Table S2 **Analysis of total aboveground biomass across the water table depth gradient on the two soil types in the two years.** Hereafter, mixed-effects models are given in R syntax so that a response is analysed as a function of (∼) fixed effects with random effects given in parentheses, as follows: Response∼fixed explanatory variables+(random effects).
(DOC)

Table S3 **Analysis of aboveground biomass yields of the species in mixture across the water table depth gradient on the two soil types.**
(DOC)

Table S4 **Analysis of relative yields of the species in mixture across the water table depth gradient on the two soil types.**
(DOC)
