# Supplementary material for: Effects of Dominance and Diversity on Productivity along Ellenberg's Experimental Water Table Gradients
Source: PLoS One. 2012 Sep 12;7(9):e43358. doi: 10.1371/journal.pone.0043358 (PMC3440424; doi:10.1371/journal.pone.0043358)
Supplement: Supporting Information S1 — Ellenberg's adjustment for incomplete cover. (DOCX) [file pone.0043358.s001.docx]

**Ellenberg’s adjustment for incomplete cover**

Ellenberg adjusted his monoculture data of 1952 when cover was below 100%. For example, if a sample (collected for a given species or mixture, at a given point on the water table gradient, in a given year and on a given soil type) yielded 100 g, but covered only 50% of the ground, Ellenberg would simply double the value to predict 200 g if cover had been 100%. (Note that in reality covers were generally high – Fig. S1 and Table S1).
