# Supplementary material for: Effects of Dominance and Diversity on Productivity along Ellenberg's Experimental Water Table Gradients
Source: PLoS One. 2012 Sep 12;7(9):e43358. doi: 10.1371/journal.pone.0043358 (PMC3440424; doi:10.1371/journal.pone.0043358)

The photograph shows the western part of the basin in 1953, filled with loam.
The view is upslope (towards north).

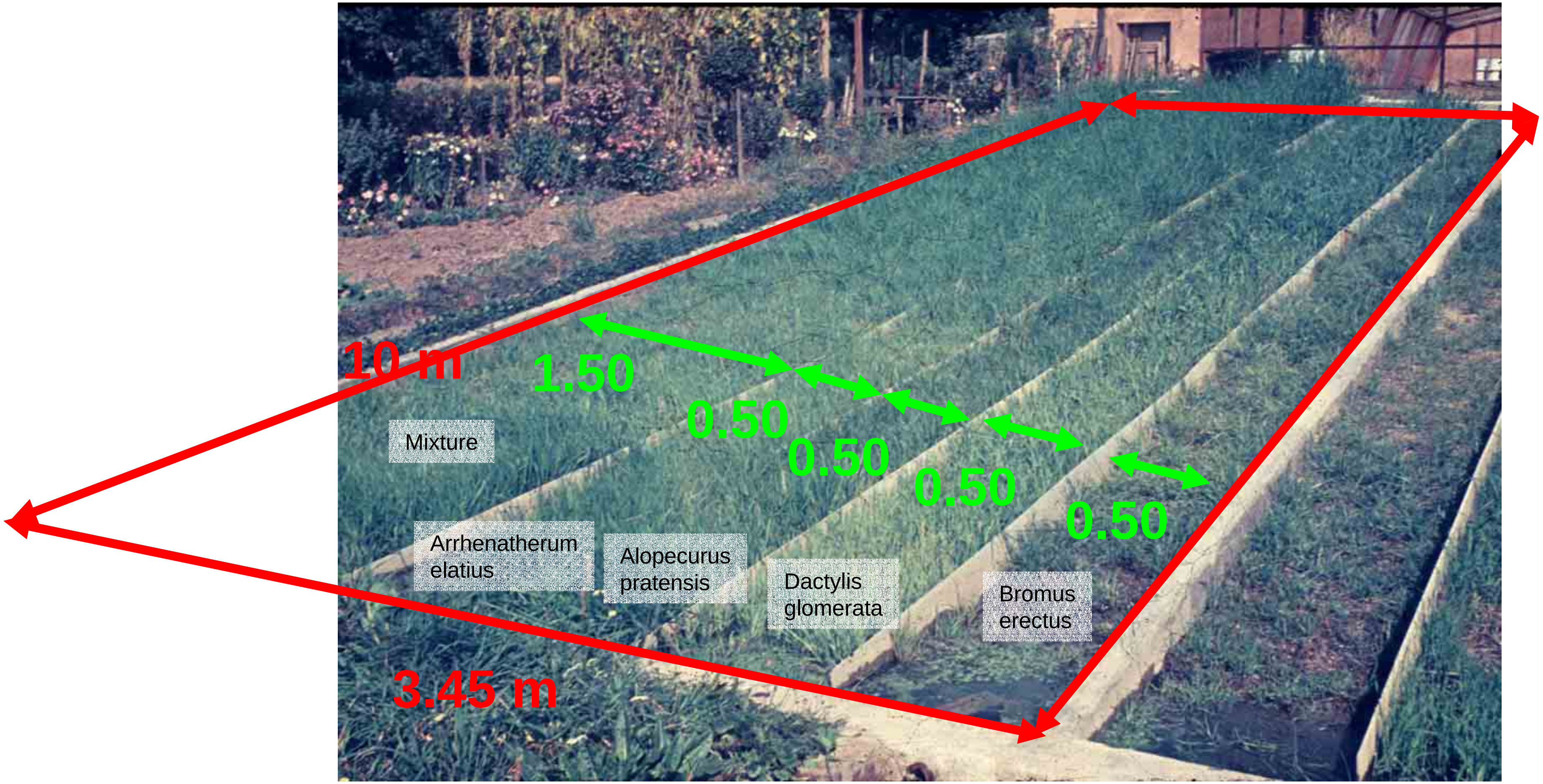

Hohenheim-Experiment-01.jpg

The photograph shows the western part of the basin in 1953, filled with loam.
The section shows the wet part of the gradient.

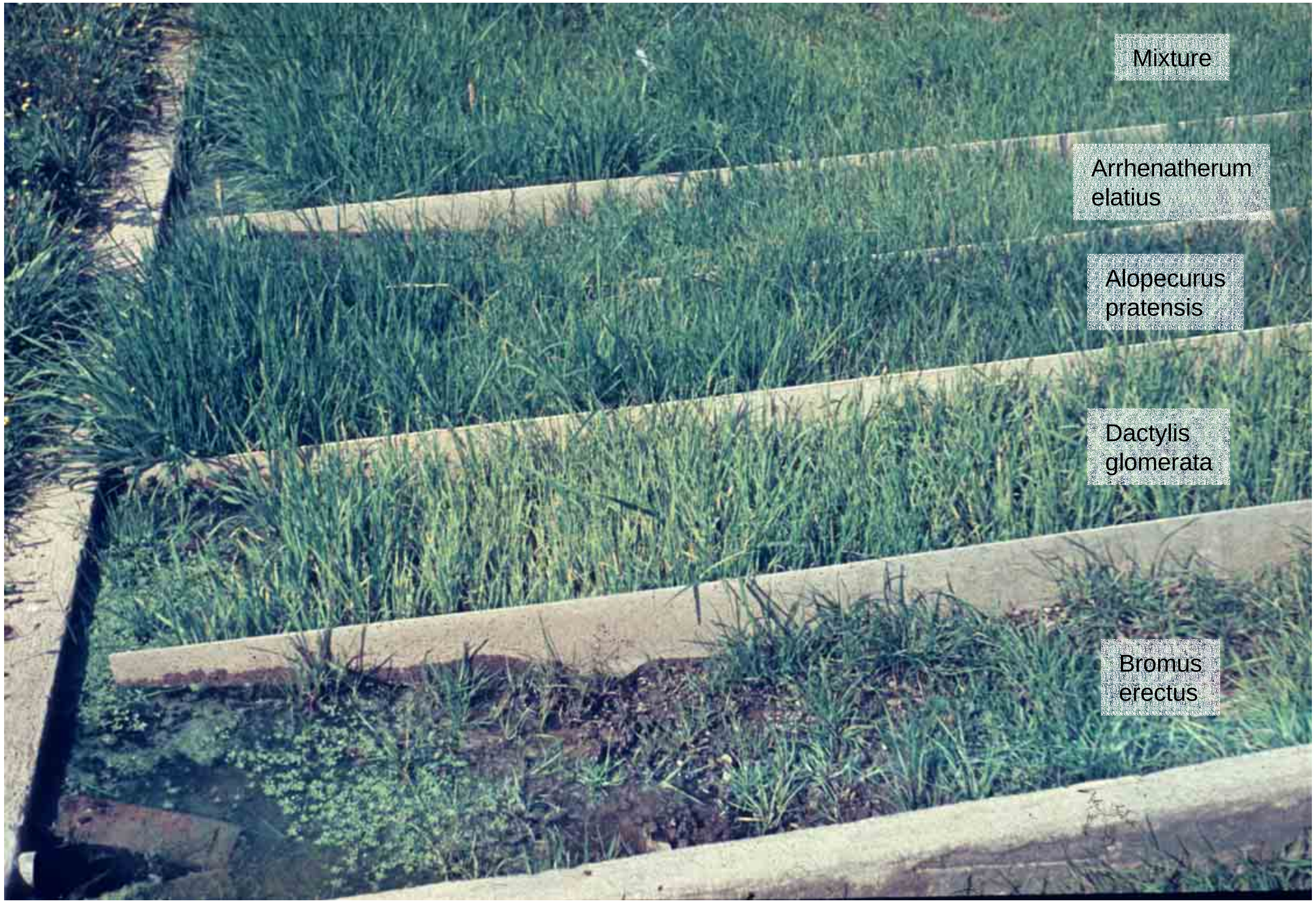

Hohenheim-Experiment-02.jpg

The photograph shows the western part of the basin in 1953, filled with loam.
The section shows the dry part of the gradient.

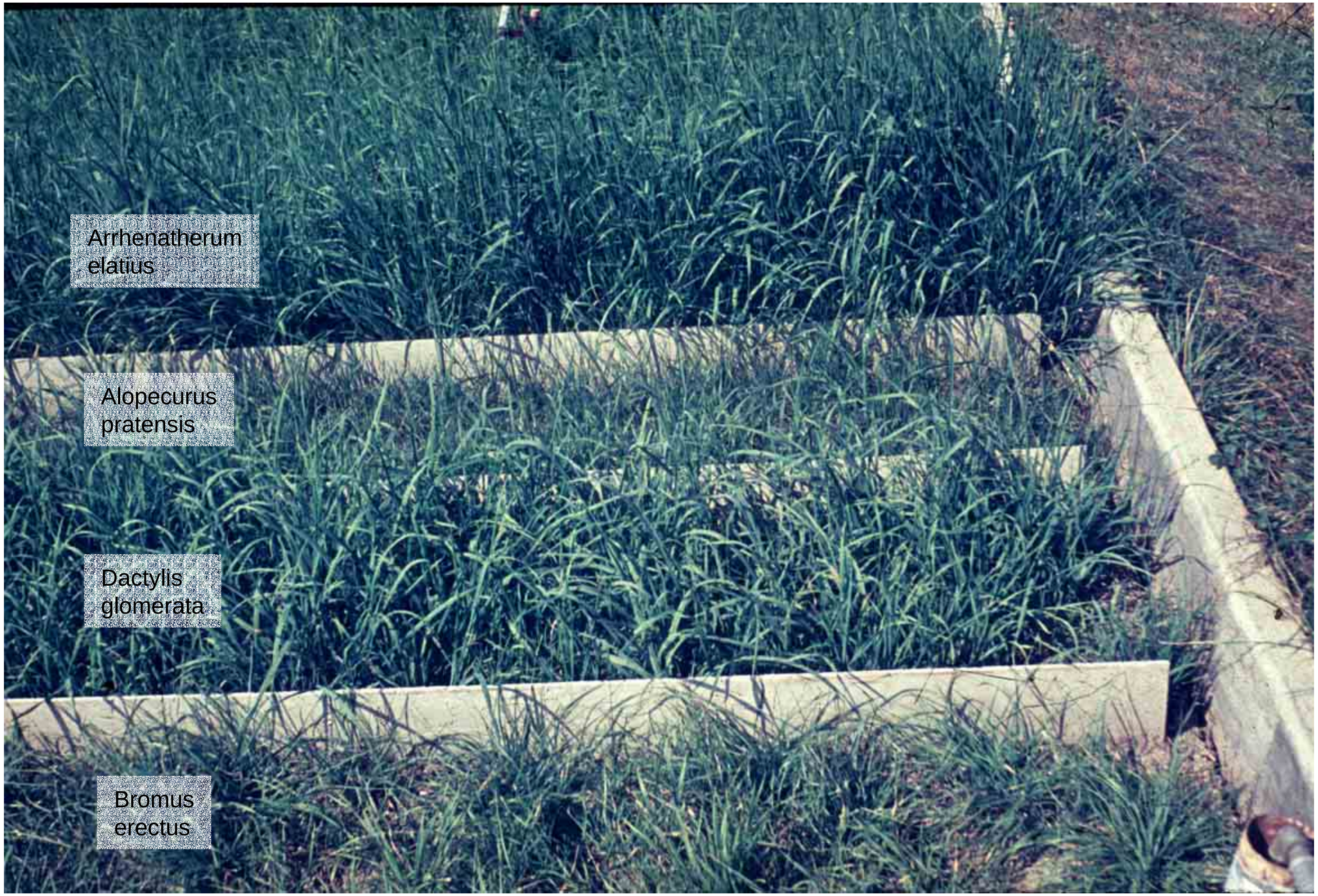

Hohenheim-Experiment-05.jpg

The photograph shows the basin in 1953.
The view is downslope (towards south).

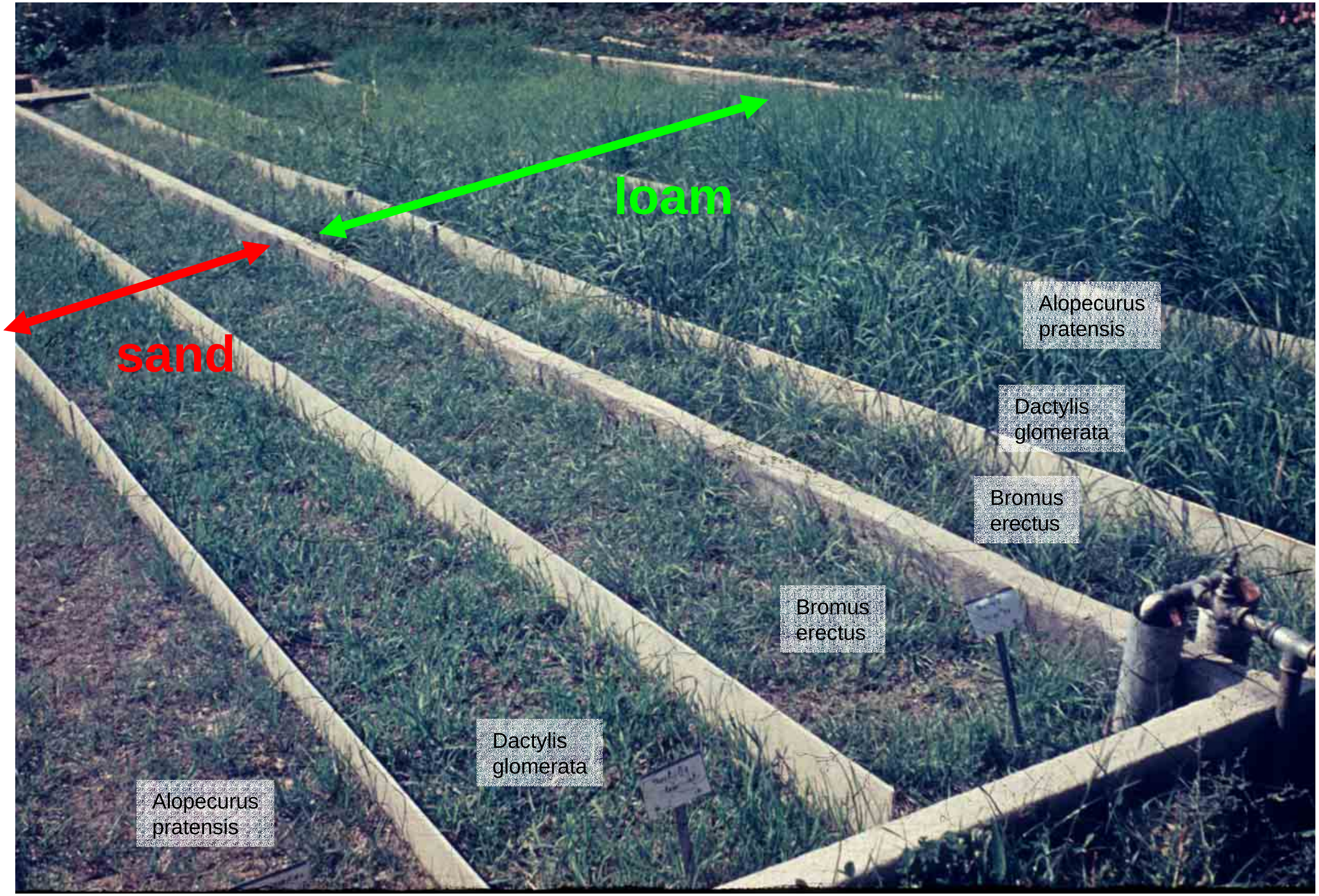

Hohenheim-Experiment-03.jpg

The photograph shows the western part of the basin in 1953, filled with loam.
The section shows the wet part of the gradient.

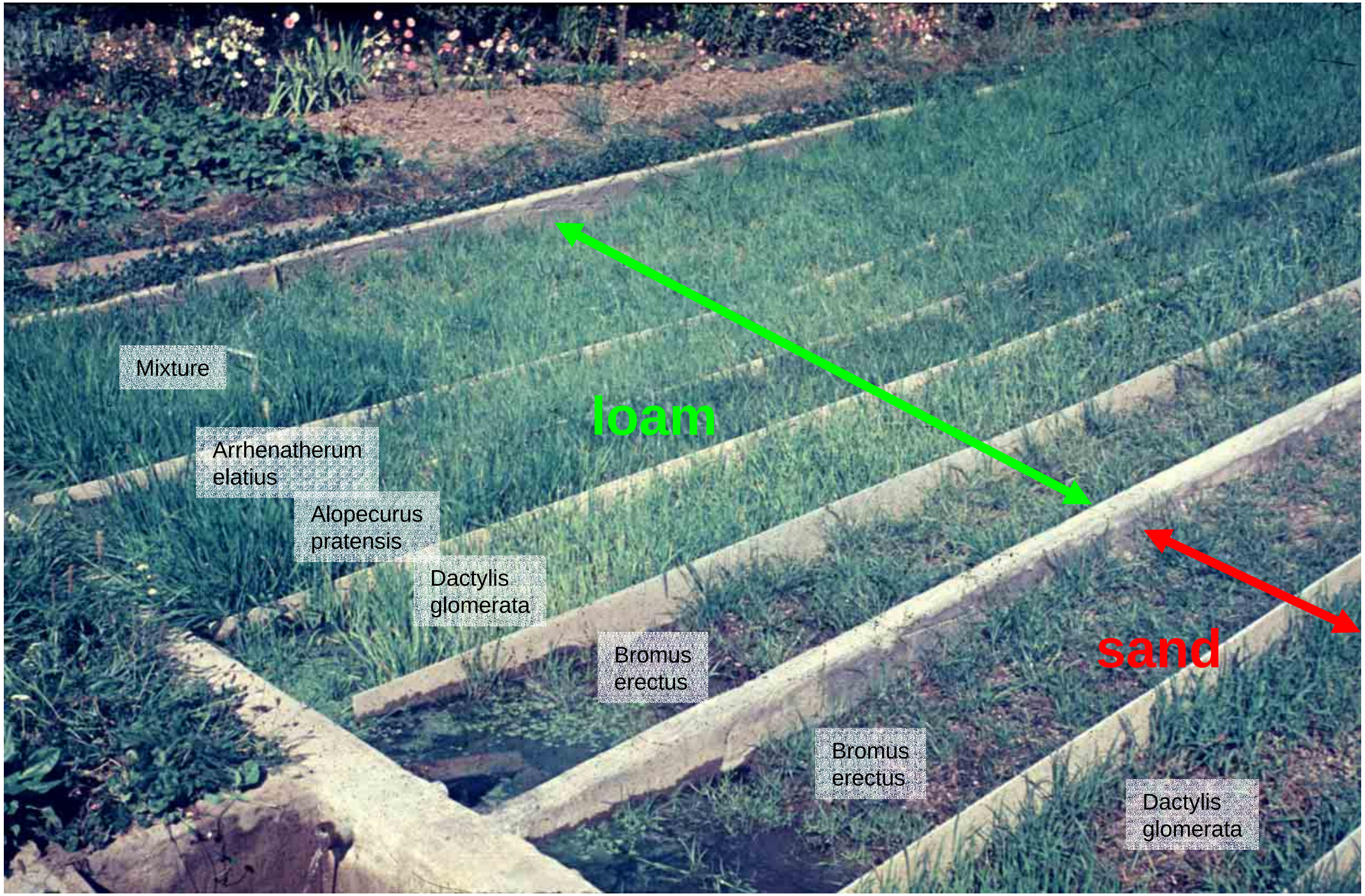

Hohenheim-Experiment-06.jpg

The photograph shows the western part of the basin in 1953, filled with loam. The view is upslope. The section shows the wet part of the gradient.

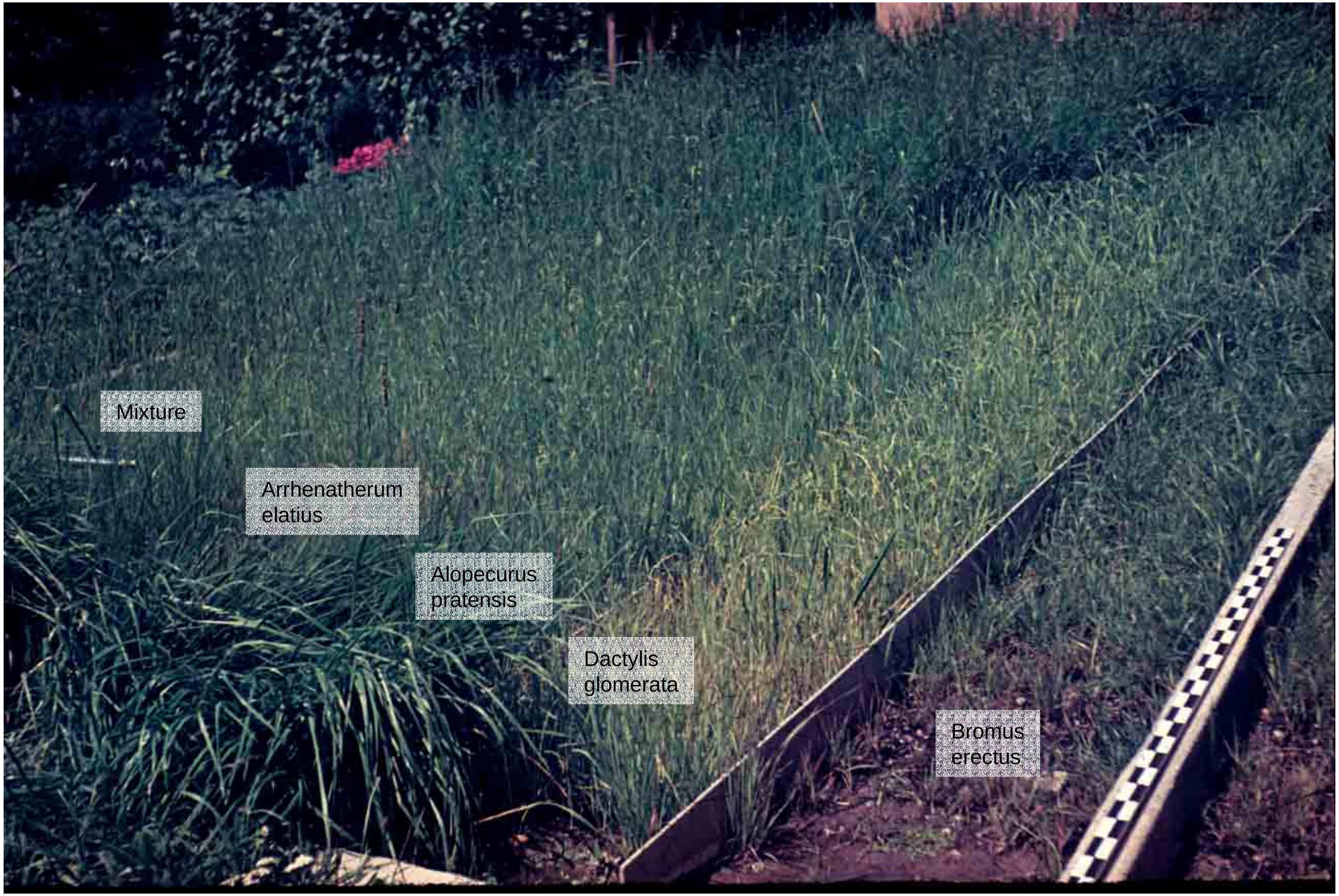

Hohenheim-Experiment-08.jpg

The photograph shows the eastern part of the basin in 1953, filled with sand. The view is upslope.

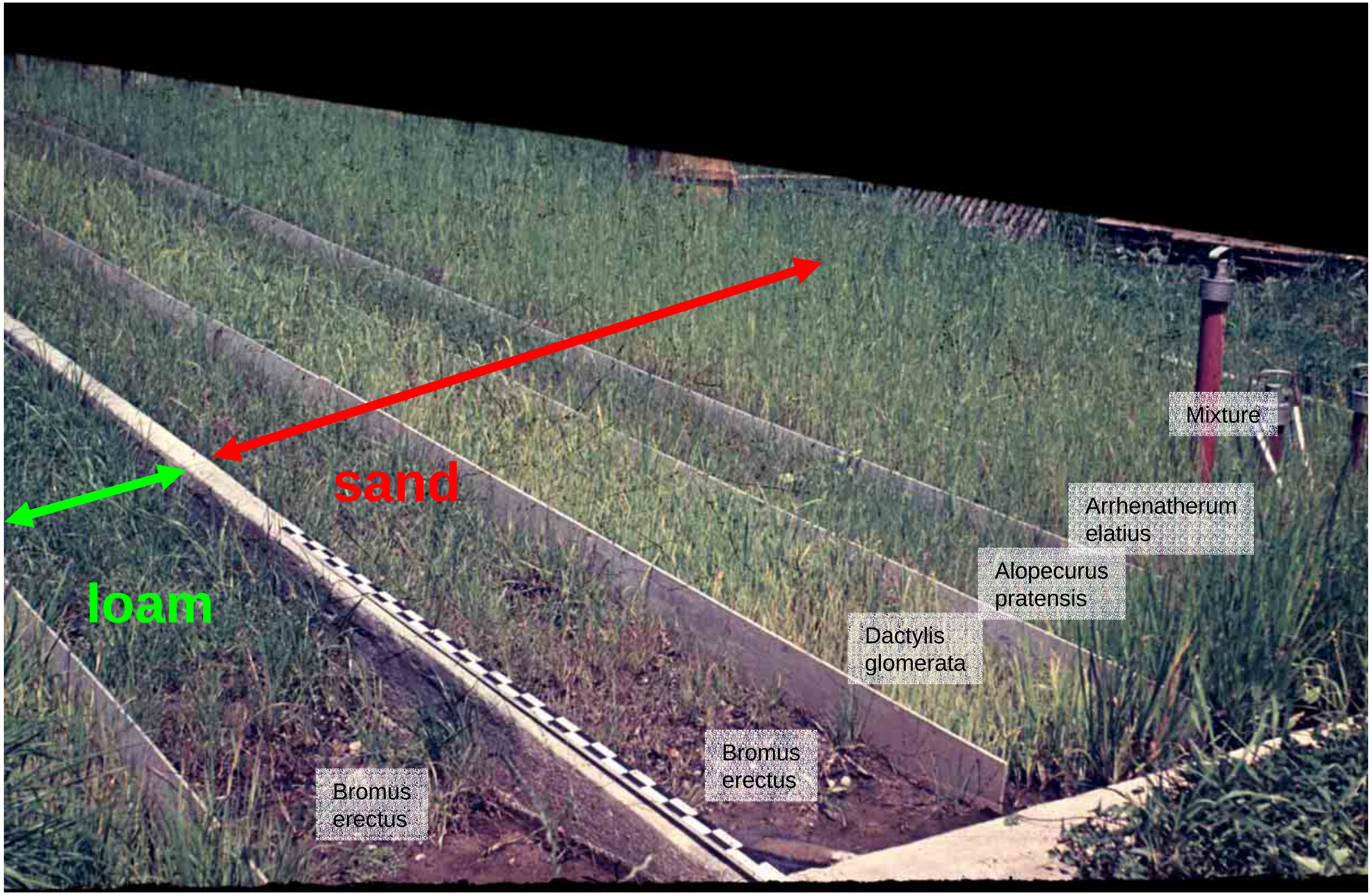

Hohenheim-Experiment-07.jpg

The photograph shows the western part of the basin in 1953, filled with loam.
The section shows the wet part of the gradient.

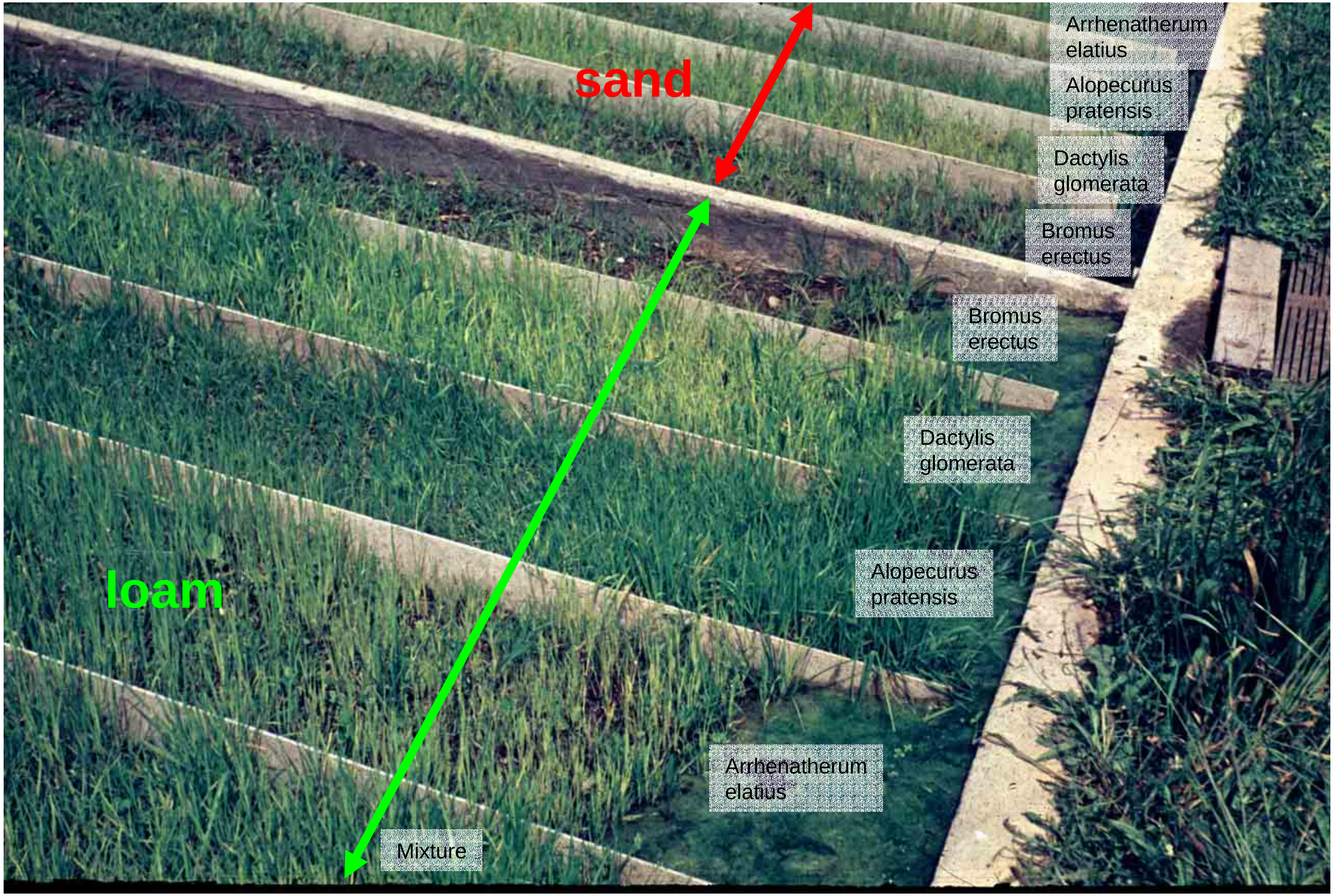

Hohenheim-Experiment-10.jpg

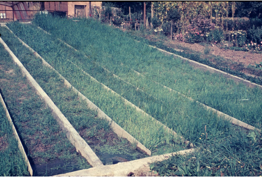

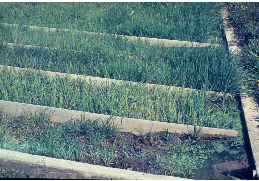

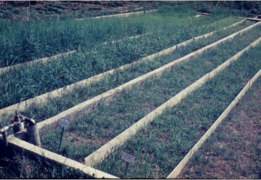

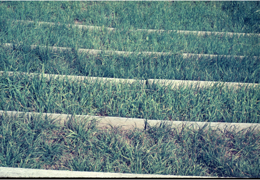

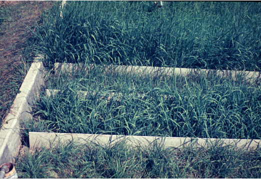

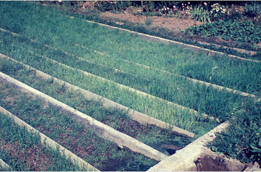

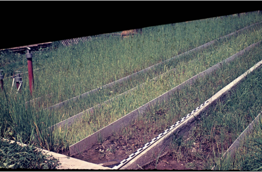

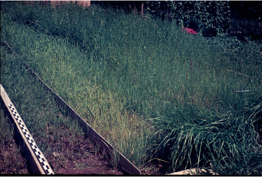

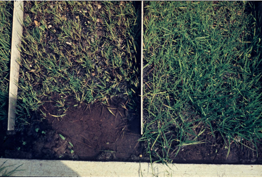

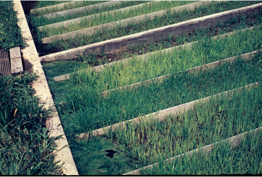

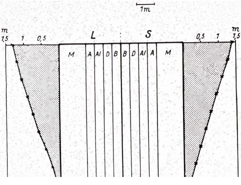

1m
m
1,5
1
0,5
L
S
M
A
Al
D
B
B
D
Al
A
M
0,5
1
m
1,5

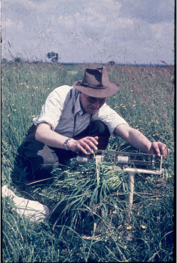

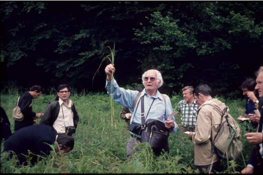

Supplement: Supporting Information S2 — Historical photographs of the Hohenheim-Experiment. (PDF) [file pone.0043358.s002.pdf]
