# Supplementary material for: Effects of Dominance and Diversity on Productivity along Ellenberg's Experimental Water Table Gradients
Source: PLoS One. 2012 Sep 12;7(9):e43358. doi: 10.1371/journal.pone.0043358 (PMC3440424; doi:10.1371/journal.pone.0043358)
Supplement: Supporting Information S3 — Metadata. (DOCX) [file pone.0043358.s003.docx]

**Metadata**

We provide two supplementary data files (including the variables detailed below):

Supplementary Information S4: EllenbergAllData.txt (raw data n=416)

Supplementary Information S5. ClimateDataHohenheim.txt (raw data n=120)

EllenbergAllData.txt

Variables:

Year: two levels: 1952 and 1953

Soil: two levels: Loam and Sand

Water: Average distance to groundwater in cm, 10 levels for 1952, 11 levels for 1953: (-5), 5, 20, 35, 50, 65, 80, 95, 110, 125, 140

Species: 6 species in 1952 and 4 species in 1953. Species 1952: Poa palustris, Festuca pratensis, Alopecurus, pratensis, Dactylis glomerata, Arrhenatherum elatius, Bromus erectus. Species 1953: Alopecurus, pratensis, Dactylis glomerata, Arrhenatherum elatius, Bromus erectus.

Mi.g: Individual yield of dried biomass in g in monocultures

Yi.g: Individual yield of dried biomass in g in mixtures

Mono.area.m2: Area of the yields in monocultures, 0.383 m in year 1952, 0.5 m in year 1953

Mix.area.m2: Area of the yields in mixtures, 1.2 m in year 1952, 1.5 m in year 1953

Div: Species richness, 6 in year 1952, 4 in year 1953

Moi.g.m2: Individual monoculture yields in m2

Yoi.g.m2: Individual mixture yields in m2

Mo.g.m2: Moi.g.m2 averaged over species by year, soil type and water level

Yo.g.m2: Yoi.g.m2 summed over species by year, soil type and water level

RYoi: Individual relative yield observed (Yoi.g.m2/ Moi.g.m2)

RYo: RYoi summed over species by year, soil type and water level

Yei.g.m2: Individual expected yield in m2 (Moi.g.m2 * RYe)

Ye.g.m2: Yei.g.m2 summed over species by year, soil type and water level

RRYo: Rescaled relative yield observed (RYoi/RYo)

deltaRYoi: Difference between relative observed yield and rescaled relative observed yield (RYoi – RRYo)

deltaRYo: deltaRYoi summed over species by year, soil type and water level

RYe: Relative yield expected in mixtures (1/Div)

deltaRYe: Difference between the rescaled relative yield observed and relative yield expected (RRYo- RYe)

RYT: Relative yield total summed over species by year, soil type and water level

level: two levels: species and community

NE: Net Effect (Yo.g.m2 - Ye.g.m2)

TICE: Trait-Independent Complementarity Effect (Mo.g.m2 * deltaRYo * Div)

SE: Selection Effect (NE – TICE)

TDCE: Trait-Dependent Complementarity Effect ((Moi.g.m2 - Mo.g.m2) * (deltaRYoi - deltaRYo) summed over species by year, soil type and water level)

DE: Diversity effect (SE – TDCE)

EllenbergClimateData.txt

Variables:

Year: two levels: 1952 and 1953

Month: twelve levels: 1 to 12 for January to December

Days.measured: Amount of measured days

Air.Temperature: Measured air temperature in Celsius

RH: Relative humidity in percent

Sunshine: Sunshine in hr

Cloud.cover: Fraction of the sky obscured by clouds

Precipitation: Percipitation in mm
