# Supplementary material for: Effects of Dominance and Diversity on Productivity along Ellenberg's Experimental Water Table Gradients
Source: PLoS One. 2012 Sep 12;7(9):e43358. doi: 10.1371/journal.pone.0043358 (PMC3440424; doi:10.1371/journal.pone.0043358)
Supplement: Supporting Information S6 — Additive partitioning of biodiversity effects. (DOCX) [file pone.0043358.s006.docx]

**Additive Partitioning of Biodiversity Effects**

The effect of biodiversity on aboveground biomass production can be quantified as a ‘net biodiversity effect’ that can then be partitioned into a ‘selection effect’ and a ‘complementarity effect’ (in an additive way so that NE = CE + SE) following Loreau and Hector (2001). The net biodiversity effect, **Δ***Y*, is the difference between the observed yield of a mixture and its expected yield under the null hypothesis that there is no selection effect or complementarity effect. When species are sown at equal density this expected value is the average of the monoculture yields for the component species, or equivalently, the sum of their expected yields (in a two species mixture sown at equal density the expected yield is simply the sum of half of the respective monoculture yields). The selection effect is measured by the covariance between the monoculture yield of species and their deviation from the expected relative yield in mixture (when species are sown at equal density the expectation is 1/S, where S is the number of species, so that the expected relative yield in a two-species mixture is 1/2). Note that because the covariance applies to the whole population of species in each mixtures rather than a sample of species the calculation is done using N (the number of species) and not the more usual N-1 (N-1 is usually the default for covariance functions in most software packages). Positive selection occurs when, on average, species with higher-than-average monoculture yields increase in relative abundance in mixtures and negative selection when species with lower-than-average yields increase in relative mixtures. Positive complementarity effects occur when increases in the relative yields of some species are not exactly compensated by decreases in others (i.e. assuming intraspecific interactions equal interspecific interactions and a zero-sum game in competition for resources). These various effects can be related by additive partition as follows:

Define, for any mixture,

*M_i_* = yield of species *i* in monoculture;

*Y_O,i_* = observed yield of species *i* in the mixture;

*$Y_{O}=\sum_{i} Y_{O,i}$* = total observed yield of the mixture;

*RY_E,i_* = expected relative yield of species *i* in the mixture, which is simply its proportion seeded or planted;

*RY_O,i_* = *Y_O,i_*/*M_i_* = observed relative yield of species *i* in the mixture;

*Y_E,i_* = *RY_E,i_M_i_* = expected yield of species *i* in the mixture;

*$Y_{E}=\sum_{i} Y_{E,i}$* = total expected yield of the mixture;

**Δ***Y* = *Y_O_* – *Y_E_* = deviation from total expected yield in the mixture;

**Δ***RY_i_* = *RY_O,i_* – *RY_E,i_* = deviation from expected relative yield of species *i* in the mixture;

*N* = number of species in the mixture.

It then follows:

$$\Delta Y=Y_{O}-Y_{E}=\sum_{i} RY_{O,i}M_{i}-\sum_{i} RY_{E,i}M_{i}=\sum_{i} \Delta RY_{i}M_{i}=N.\overline{\Delta RY}.\overline{M}+N.\mathrm{cov}(\Delta RY,M)$$

In this equation, $N.\overline{\Delta RY}.\overline{M}$ measures the complementarity effect, and $N.\mathrm{cov}(\Delta RY,M)$ measures the selection effect. Note that this approach is a generalisation of the widely-used relative yield total (*RYT*) approach in plant population biology and intercropping and, more recently, the proportional deviation from expected value (*D*) approach devised by Loreau (Oikos 1998).
