# Supplementary figures and images for: Effects of Dominance and Diversity on Productivity along Ellenberg's Experimental Water Table Gradients
Source: PLoS One. 2012 Sep 12;7(9):e43358. doi: 10.1371/journal.pone.0043358 (PMC3440424; doi:10.1371/journal.pone.0043358)

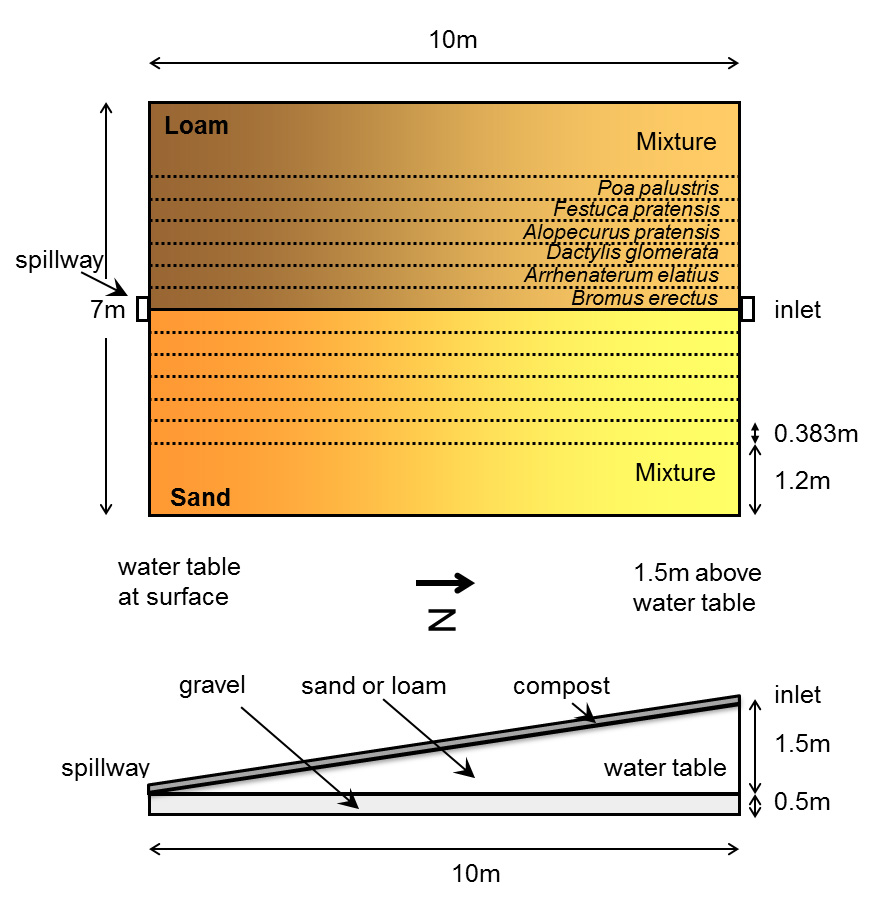

Supplement: Figure S1 — Schematic diagram of Ellenberg's water table depth gradient experiment in 1952. (TIF) [file pone.0043358.s007.tif]

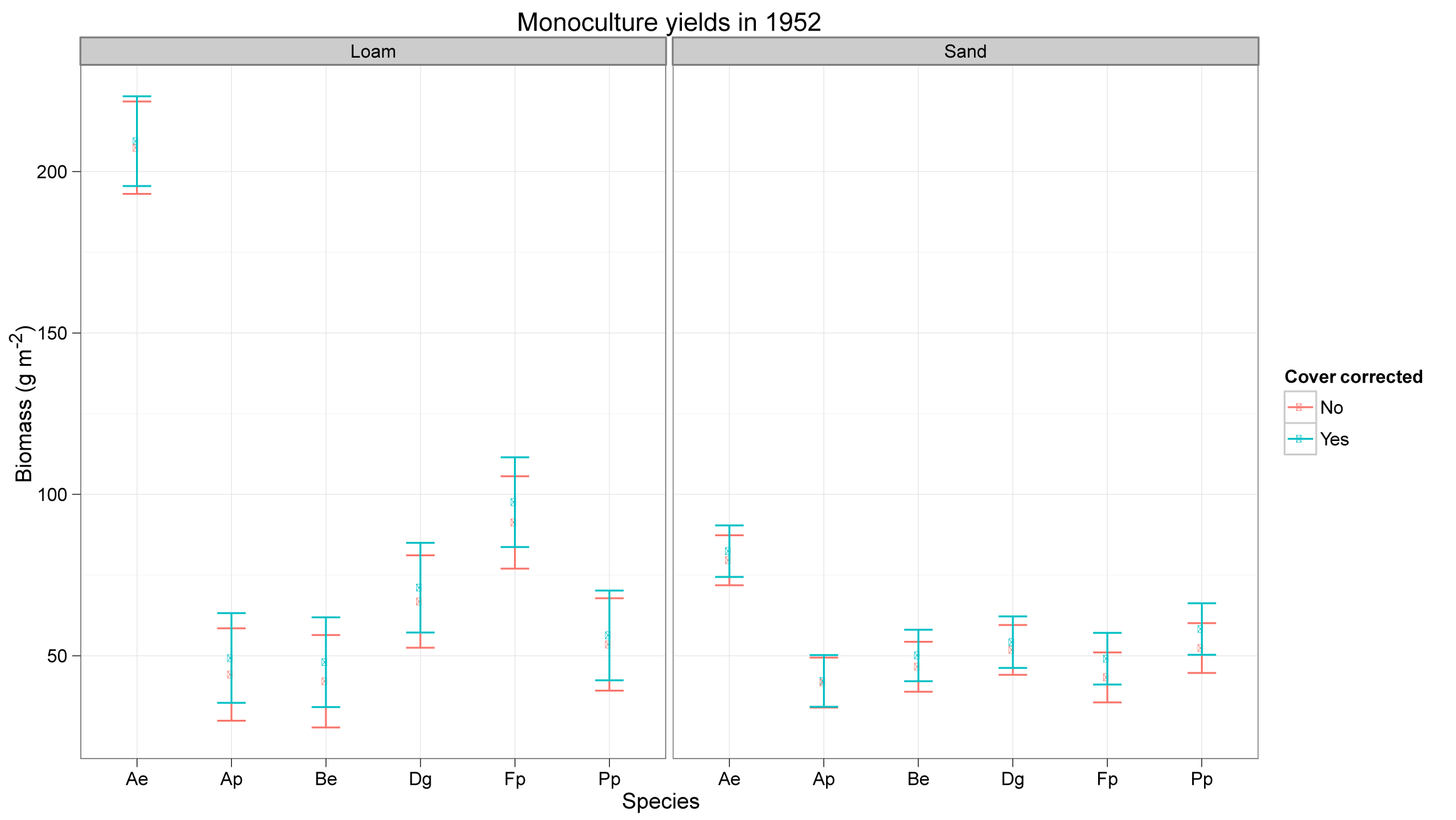

Supplement: Figure S2 — Means biomass for the monocultures of the 6 species used in the 1952 run of the experiment. Green symbols show Ellenberg's data as adjusted for cover levels below 100% and red symbols show the same mean values allowing for the observed level of cover (i.e. undoing Ellenberg's changes). (TIF) [file pone.0043358.s008.tif]

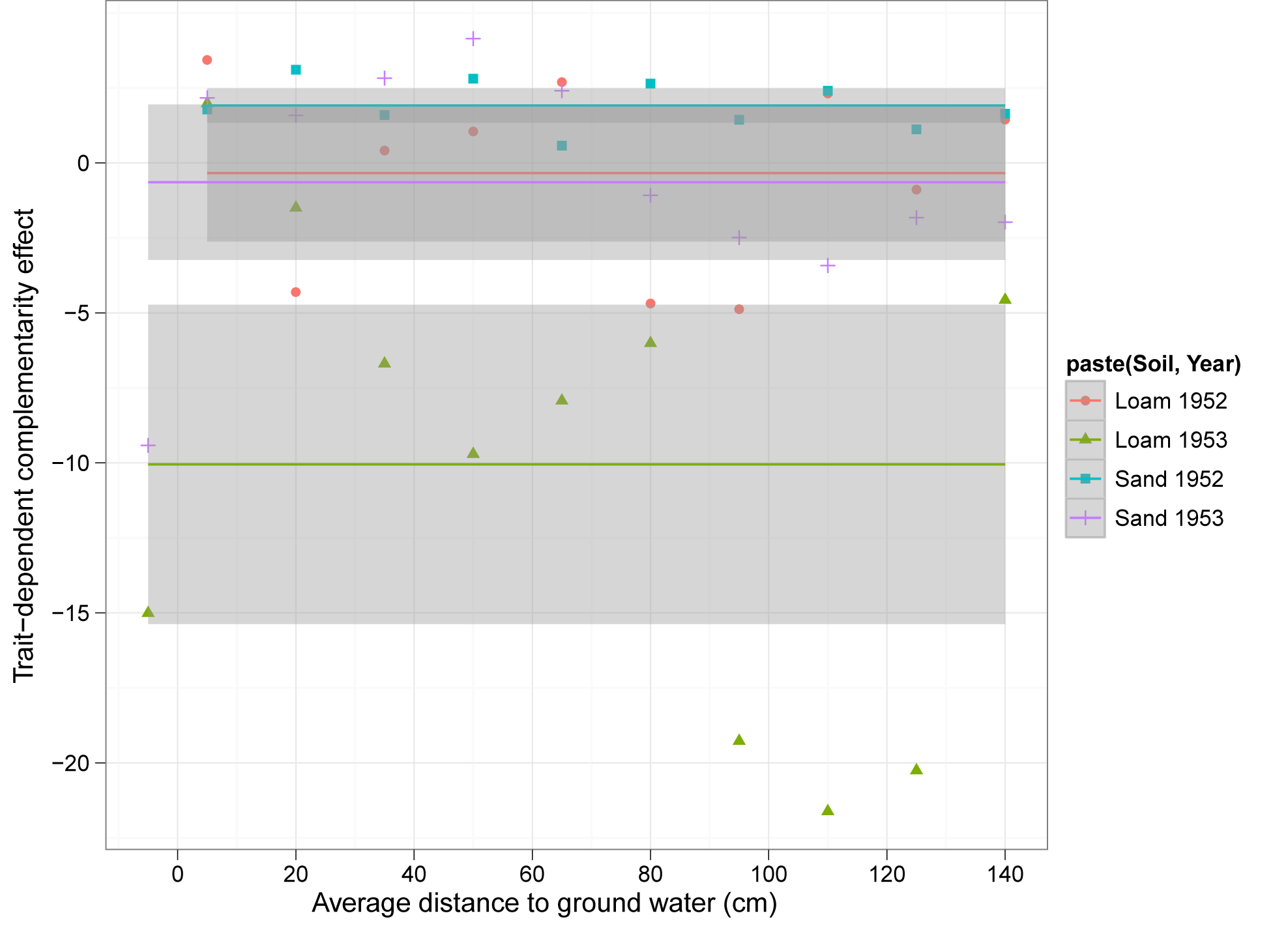

Supplement: Figure S3 — Results of the analysis of the effect of the water table depth on the strength and direction of the trait-dependent complementarity effect on sand and loam soils in 1952 and 1953. The lines are slopes from the mixed-effects model with their 95% confidence intervals (shaded). (TIF) [file pone.0043358.s009.tif]

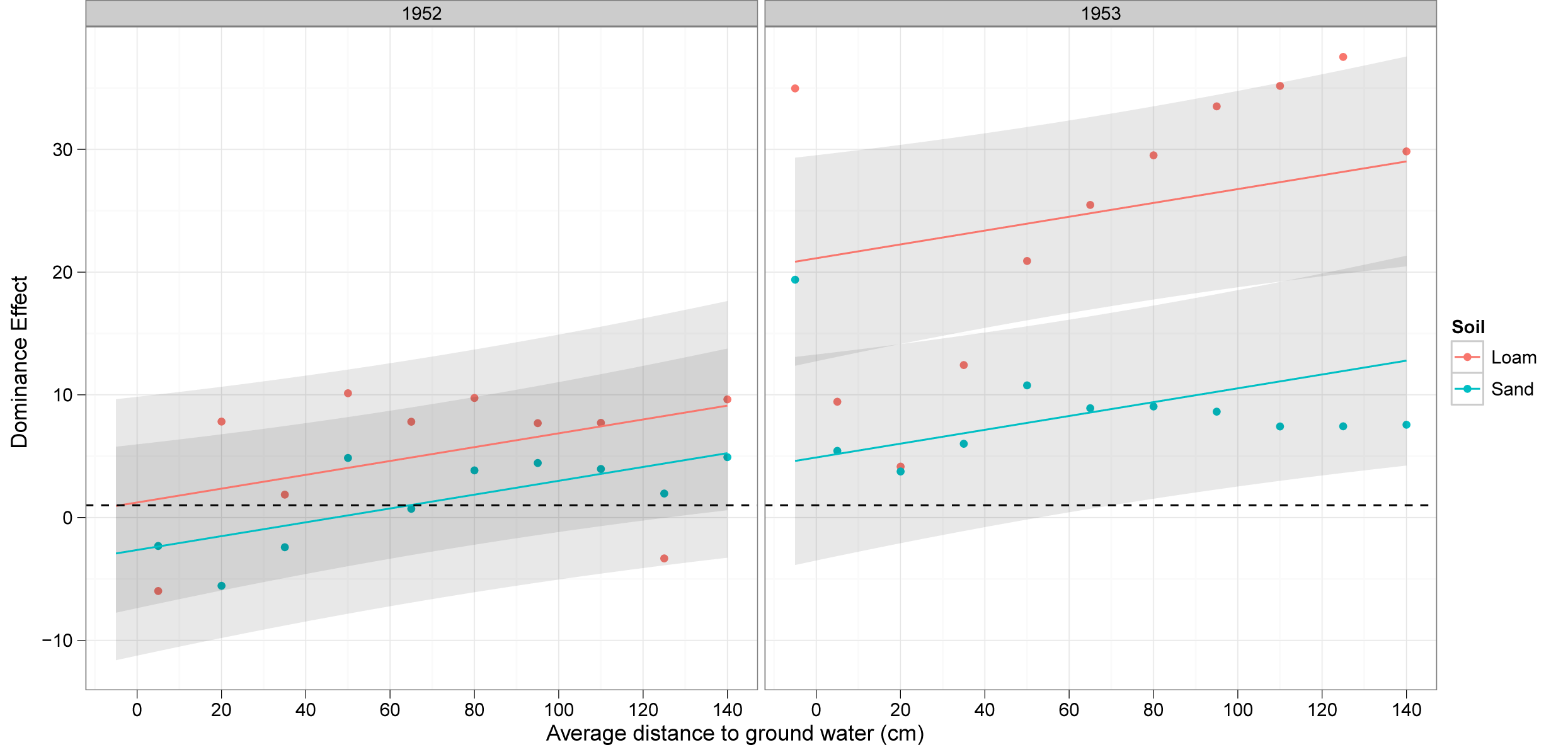

Supplement: Figure S4 — Results of the analysis of the effect of the water table depth on the strength and direction of dominance effect on sand and loam soils in 1952 and 1953. The lines are slopes from the mixed-effects model with their 95% confidence intervals (shaded). (TIF) [file pone.0043358.s010.tif]

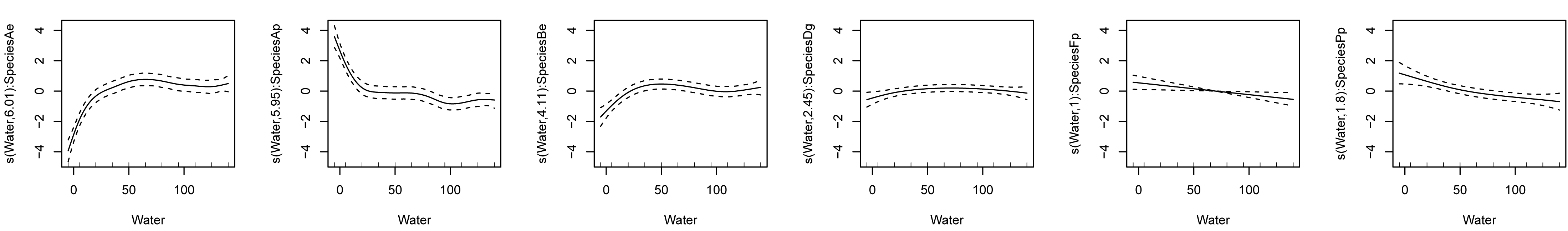

Supplement: Figure S5 — Estimated smoothing curves for the yields of individual species across the experimental water table depth gradient. The solid line is the smoother and the dotted lines are 95% point-wise confidence bands. Note that the smoother is centred around zero. (TIF) [file pone.0043358.s011.tif]

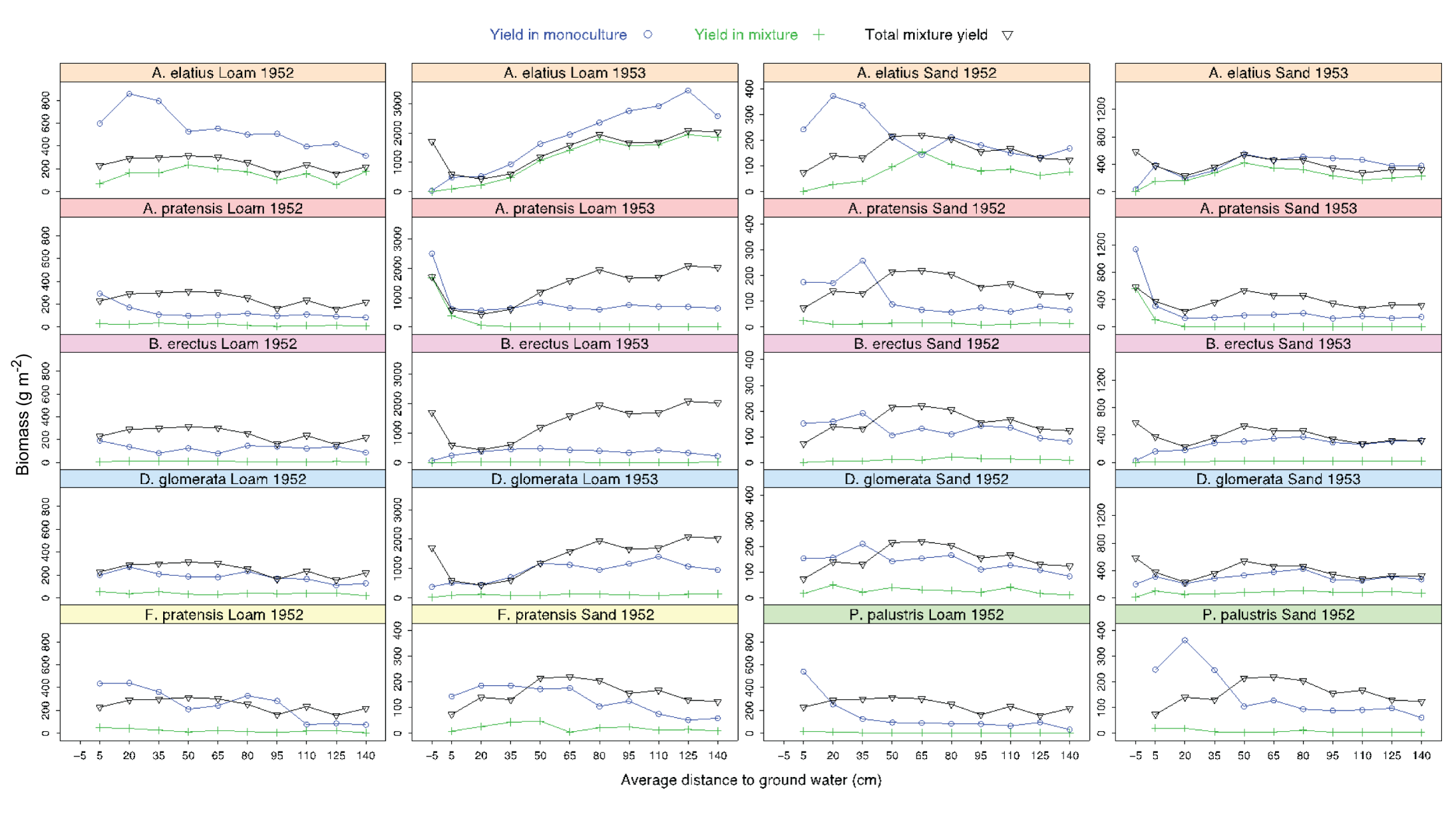

Supplement: Figure S6 — Dry biomass. Aboveground biomass of species in monoculture (blue) and mixture (green) on the two soil types in the two experimental years together with the total community aboveground biomass (black). Note that the black symbols within each column of panels are the same and are the sum of the green symbols. (TIF) [file pone.0043358.s012.tif]

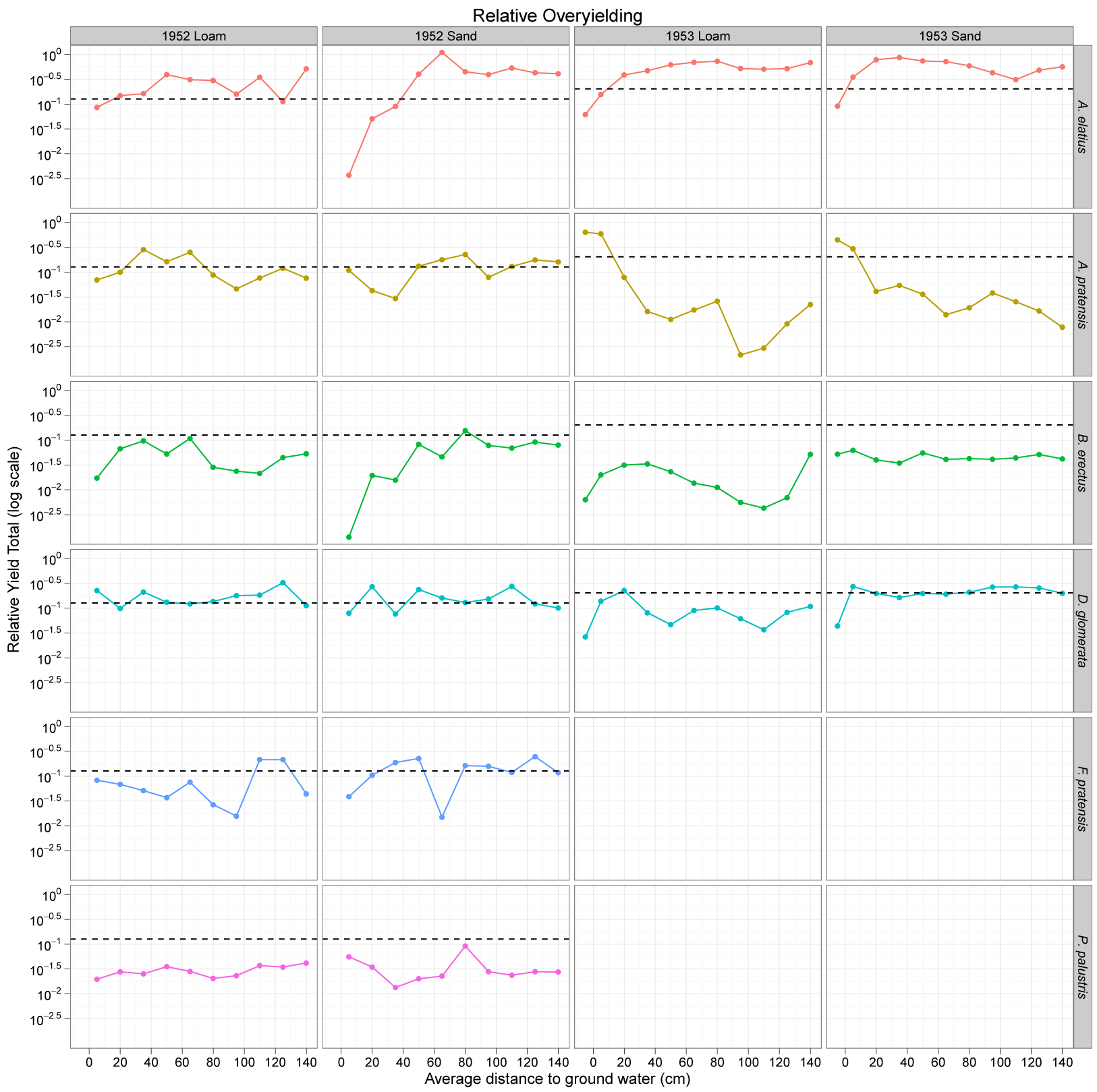

Supplement: Figure S7 — Relative yields of six different species across the water table depth gradient on two soil types in two years. Note the absence of two species in 1953. (TIF) [file pone.0043358.s013.tif]

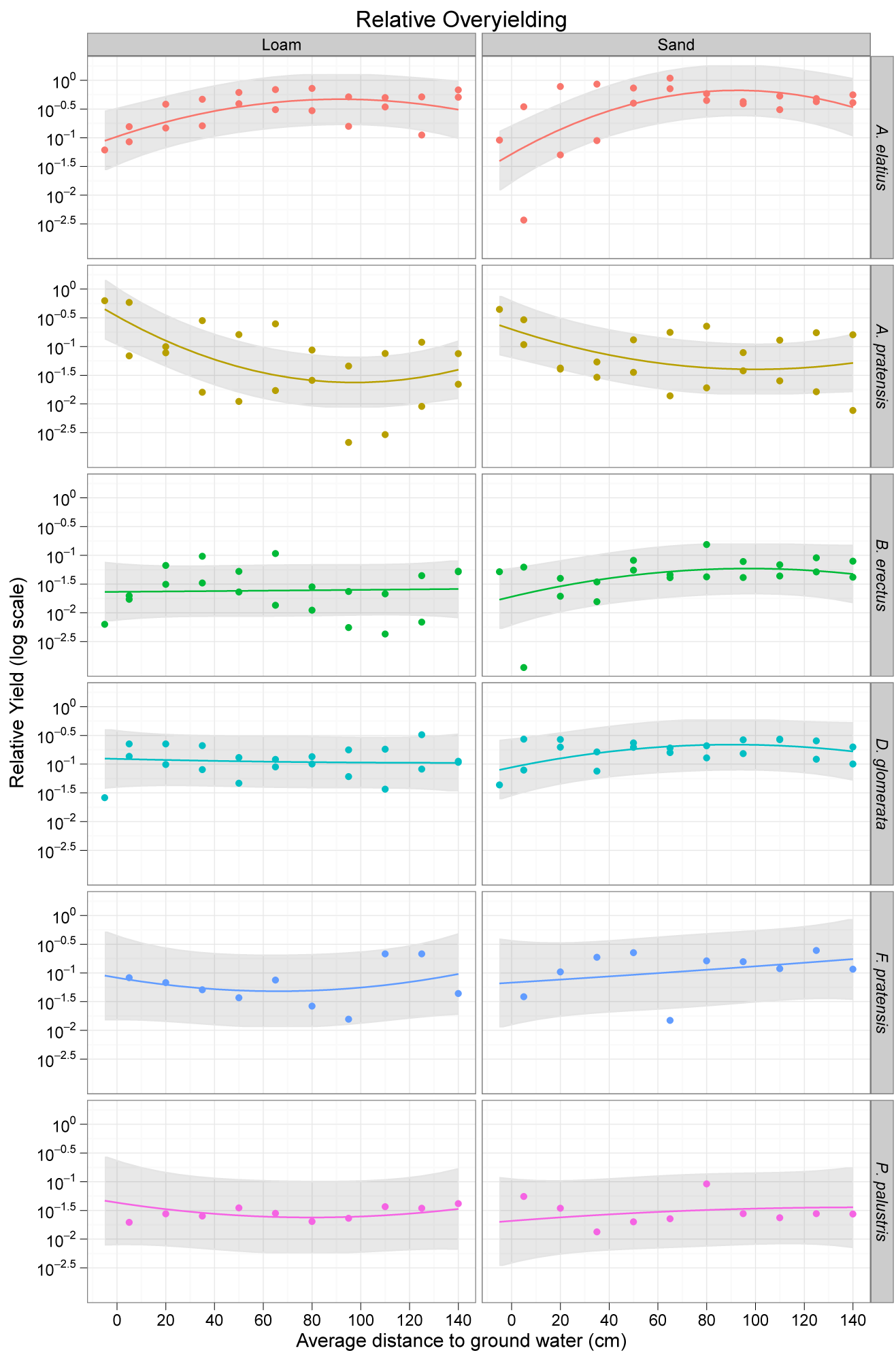

Supplement: Figure S8 — Results of the mixed-effects model analysis of the relative yields of individual species across the experimental water table depth gradient. The curves are back-transformed slopes from the mixed-effects model with their 95% confidence intervals (shaded). (TIF) [file pone.0043358.s014.tif]

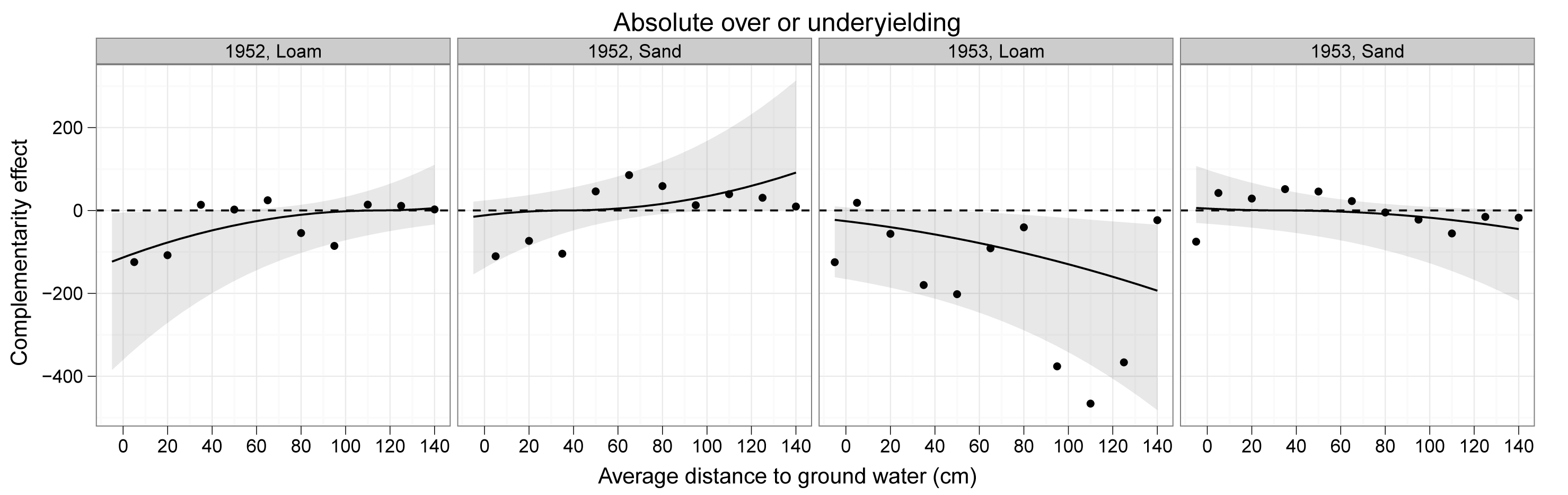

Supplement: Figure S9 — Results of the analysis of the effect of the water table depth on the strength and direction of the trait-independent complementarity effect on sand and loam soils in 1952 and 1953. The curves are back-transformed slopes from the mixed-effects model with their 95% confidence intervals (shaded). The negative Complementarity Effect on loam in 1953 is mainly driven by increasing dominance by Arrhenatherum elatius as depth to the water table increased. (TIF) [file pone.0043358.s015.tif]

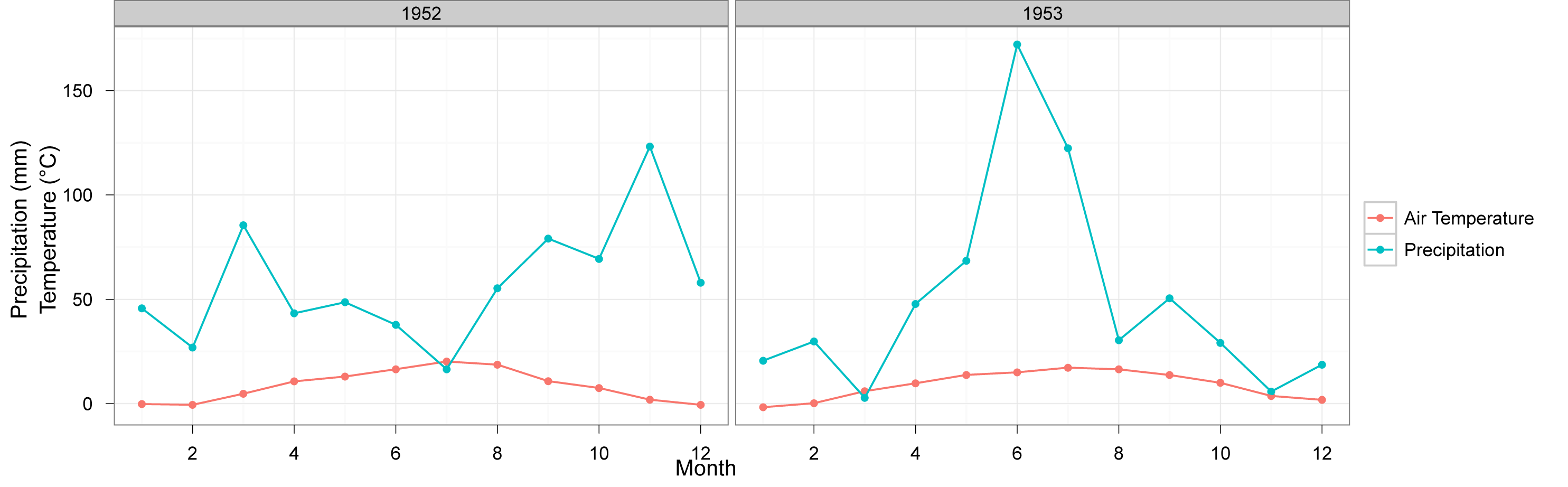

Supplement: Figure S10 — The climate data from the weather station in Hohenheim clearly showing that 1953 had a very wet summer. (TIF) [file pone.0043358.s016.tif]
