# Supplementary material for: Effects of Dominance and Diversity on Productivity along Ellenberg's Experimental Water Table Gradients
Source: PLoS One. 2012 Sep 12;7(9):e43358. doi: 10.1371/journal.pone.0043358 (PMC3440424; doi:10.1371/journal.pone.0043358)
Supplement: Table S1 — Available cover data of the monocultures in 1952 with median cover, cover corrected biomass mean (se) in g and the uncorrected biomass mean (se) in g within species and by soil type. (DOC) [file pone.0043358.s017.doc]

Table S1. This table is showing the available cover data of the monocultures in 1952 with median cover, cover corrected biomass mean (se) in g and the uncorrected biomass mean (se) in g within species and by soil type.

| Soil | Species | Cover  median | Cover corrected biomass  mean (se) in g | Cover uncorrected biomass mean (se) in g |
| --- | --- | --- | --- | --- |
| Loam | P. palustris | 0.95 | 56.30 (13.9) | 53.50 (14.30) |
|  | F. pratensis | 0.90 | 97.60 (13.9) | 91.30 (14.30) |
|  | A. pratensis | 0.90 | 49.30 (13.9) | 44.20 (14.30) |
|  | D. glomerata | 0.93 | 71.10 (13.9) | 66.80 (14.30) |
|  | A. elatius | 1.00 | 209.40 (13.9) | 207.40 (14.30) |
|  | B. erectus | 0.83 | 48.00 (13.9) | 42.10 (14.30) |
| Sand | P. palustris | 0.95 | 58.3 (7.99) | 52.4 (7.73) |
|  | F. pratensis | 0.90 | 49.1 (7.99) | 43.3 (7.73) |
|  | A. pratensis | 1.00 | 42.2 (7.99) | 41.7 (7.73) |
|  | D. glomerata | 1.00 | 54.2 (7.99) | 51.8 (7.73) |
|  | A. elatius | 1.00 | 82.4 (7.99) | 79.6 (7.73) |
|  | B. erectus | 0.93 | 50.1 (7.99) | 46.6 (7.73) |
