# Supplementary material for: Effects of Dominance and Diversity on Productivity along Ellenberg's Experimental Water Table Gradients
Source: PLoS One. 2012 Sep 12;7(9):e43358. doi: 10.1371/journal.pone.0043358 (PMC3440424; doi:10.1371/journal.pone.0043358)
Supplement: Table S2 — Analysis of total aboveground biomass across the water table depth gradient on the two soil types in the two years. Hereafter, mixed-effects models are given in R syntax so that a response is analysed as a function of (∼) fixed effects with random effects given in parentheses, as follows: Response∼fixed explanatory variables+(random effects). (DOC) [file pone.0043358.s018.doc]

Table S2. Analysis of total aboveground biomass across the water table depth gradient on the two soil types in the two years. Hereafter, mixed-effects models are given in R syntax so that a response is analysed as a function of (~) fixed effects with random effects given in parentheses, as follows: Response ~ fixed explanatory variables + (random effects).

model1 <- lmer( log(Yo.g.m2) ~ Soil*Water*Year+(1|Gradient), method= "ML", data= Community, na.action=na.omit)

model2 <- lmer( log(Yo.g.m2) ~ Soil+Water+Year+Soil:Water+Soil:Year+Year:Water +(1|Gradient), method= "ML", data= Community, na.action=na.omit)

anova(model1, model2)

| Model | Df | BIC |
| --- | --- | --- |
| model2 | 9 | 62.618 |
| model1 | 10 | 54.998 |
