# Supplementary material for: Effects of Dominance and Diversity on Productivity along Ellenberg's Experimental Water Table Gradients
Source: PLoS One. 2012 Sep 12;7(9):e43358. doi: 10.1371/journal.pone.0043358 (PMC3440424; doi:10.1371/journal.pone.0043358)
Supplement: Table S3 — Analysis of aboveground biomass yields of the species in mixture across the water table depth gradient on the two soil types. (DOC) [file pone.0043358.s019.doc]

Table S3. Analysis of aboveground biomass yields of the species in mixture across the water table depth gradient on the two soil types.

model1 <- lmer(log(Yoi.g.m2) ~ Species*(Water+I(Water^2))+Soil*(Water+I(Water^2))+(1|Gradient)+(1|Strip), method= "ML", data= Species)

model2 <- lmer(log(Yoi.g.m2) ~ Species*(Water+I(Water^2))+Soil*Water+(1|Gradient)+(1|Strip), method= "ML", data= Species)

model3 <- lmer(log(Yoi.g.m2) ~ Species*(Water+I(Water^2))+Soil+(1|Gradient)+(1|Strip), method= "ML", data= Species)

model4 <- lmer(log(Yoi.g.m2) ~ Species*(Water+I(Water^2))+(1|Gradient)+(1|Strip), method= "ML", data= Species)

model5 <- lmer(log(Yoi.g.m2) ~ Species+(Water+I(Water^2))+(1|Gradient)+(1|Strip), method= "ML", data= Species)

anova(model1, model2, model3, model4, model5)

| Model | Df | BIC |
| --- | --- | --- |
| model5 | 11 | 658.57 |
| model4 | 21 | 576.06 |
| model3 | 22 | 580.30 |
| model2 | 23 | 583.37 |
| model1 | 24 | 586.57 |
