# Supplementary material for: Effects of Dominance and Diversity on Productivity along Ellenberg's Experimental Water Table Gradients
Source: PLoS One. 2012 Sep 12;7(9):e43358. doi: 10.1371/journal.pone.0043358 (PMC3440424; doi:10.1371/journal.pone.0043358)
Supplement: Table S4 — Analysis of relative yields of the species in mixture across the water table depth gradient on the two soil types. (DOC) [file pone.0043358.s020.doc]

Table S4. Analysis of relative yields of the species in mixture across the water table depth gradient on the two soil types.

model1 <- lmer(log(RYoi) ~ Soil*Species*(Water+I(Water^2))+(1|Gradient)+(1|Strip), method= "ML", data= Species)

model2 <- lmer(log(RYoi) ~ Soil*Species*Water+Soil*(Water+I(Water^2))+Species*(Water+I(Water^2))+(1|Gradient)+(1|Strip), method= "ML", data= Species)

model3 <- lmer(log(RYoi) ~ Soil*Species+Soil*(Water+I(Water^2))+Species*(Water+I(Water^2))+(1|Gradient)+(1|Strip), method= "ML", data= Species)

anova(model1, model2, model3)

| Model | Df | BIC |
| --- | --- | --- |
| model3 | 29 | 583.01 |
| model2 | 34 | 605.92 |
| model1 | 39 | 632.00 |
